# Experimental Evaluation of UR5e Collaborative Robot Force Control in Low-Force Applications

**DOI:** 10.3390/s26051709

**Published:** 2026-03-08

**Authors:** Roman Trochimczuk, Adam Wolniakowski, Michał Ostaszewski, Andrzej Burghardt, Piotr Borkowski

**Affiliations:** 1 Department of Automatic Control and Robotics, Faculty of Electrical Engineering, Bialystok University of Technology, 15-351 Bialystok, Poland; a.wolniakowski@pb.edu.pl (A.W.); m.ostaszewski@pb.edu.pl (M.O.); 2 Department of Applied Mechanics and Robotics, Faculty of Mechanical Engineering and Aeronautics, Rzeszów University of Technology, 35-959 Rzeszów, Poland; andrzejb@prz.edu.pl; 3 Department of Biomechatronics, Faculty of Mechanical Engineering, Bialystok University of Technology, 15-351 Bialystok, Poland; p.borkowski@pb.edu.pl

**Keywords:** UR5e, cobots, low-force application, measurement of force, force/torque sensor

## Abstract

This article presents the findings of experimental research conducted to assess the stability of the force mode of the UR5e cobot from Universal Robots in the low-force range, from 1 N to 10 N. The set values of the robot’s forces and the physically measured values were verified by an OptoForce Hex six-axis Force/Torque sensor attached to the robot’s wrist, additionally coupled with an end-effector specially designed for research purposes. The results were recorded using proprietary software developed in the LabVIEW environment and a configured test lab station with a UR5e cobot. Three experimental tests were performed, in which the parameters of the effective force were measured while varying (1) the position of the task in the workspace of the robot, (2) the position and the level of force, and (3) the controller parameters of the force mode. The results of the experiments were compiled and presented in tables containing descriptions of, among other parameters, the following: the mean forces and their standard deviation; the mean maximum forces and its standard deviation; the mean root mean square error and its standard deviation; the mean absolute error and its standard deviation; the mean rate of force and its standard deviation; and the mean overshoot and its standard deviation. The findings of Experiment 1 demonstrated that when a setpoint of 10 N was employed, the UR5e cobot yielded an actual mean force ranging from 8.95 N to 13.26 N within the workspace plane. Experiment 2 showed that the average deviation from the set value within the 1–10 N range was approximately 0.38 N, with a maximum deviation of 0.61 N occurring at the limits of the working space. Experiment 3 showed that for the force range of 1–4 N, the best controller settings are Gain = 0.5 and Damping = 0.7; for the force range of 5–7 N: Gain = 1.0 and Damping = 0.6; and for the force range of 8–10 N: Gain = 2.0 and Damping = 0.8. Polynomial regression models were developed for each positioning scenario that can be used when making decisions regarding practical applications of the low-force mode.

## 1. Introduction

Flexible Manufacturing Systems (FMSs) are primarily designed for the production of short series of products with diverse functional characteristics, with minimal production line changeover time [1]. The basis for the design of flexible solutions are mainly CNC machines, working in conjunction with various classes of industrial robots. The aforementioned components of the production system are interconnected to form homogeneous manufacturing systems, with the additional use of automatic interoperational transport systems, Automated Storage and Retrieval Systems (ASRSs), and superior computer control systems in the form of a Programmable Logic Controller (PLC) or industrial control computers integrated with Manufacturing Execution System/Material Requirements Planning—MES/MRP class systems [2]. Despite the high degree of automation of such solutions, in many cases, certain manufacturing activities are still performed by operators, who often have to work in close proximity to various technical devices, including robots. The degree of cooperation between the operator and the robot can vary, ranging from coexistence, through sequential cooperation and cooperation, to collaboration [3]. The solution to the necessity for collaboration between operators and industrial robots is cobots (collaborative robots) [4,5]. These robots are designed specifically to perform the aforementioned types of cooperation, ensuring a high level of safety during operation and coexistence of robot/operator subsystems in an industrial environment, obviating the requirement for additional safety guards.

Currently, the portfolios of the world’s leading robot manufacturers all include cobots (collaborative robots). One of the first cobot manufacturers, and widely considered the most significant, is the European company Universal Robots, based in Denmark, which manufactures cobots from the UR series [6]. The incorporation of force/torque sensors within the robot’s architecture enables the UR5e to operate in scenarios where safety is of the utmost importance. These applications include the inspection of processed objects, the facilitation of motion programming with compensation for object mass, precision assembly operations, and grinding, polishing, and deburring processes. Each of the above applications requires the robot user to define the force value, its direction of action, and the appropriate combination of these parameters, while also defining the movement path. In many cases of industrial robot applications with force modes, such robots exert a very good force on the processed objects, above 10 N, which can be considered a certain threshold value for their proper operation.

When analysing the results of research on the application of robots in force-based applications, they can mainly be reduced to articles referring to the construction of new robotic solutions based on parallel robot structures, as well as robots with open kinematic chains, in the following applications: assembly, grinding, polishing, and inspection of processed objects. In the majority of cases, the primary scientific work focuses on the implementation of novel force control strategies within the context of kinematic chains. For instance, in article [7], the authors analyse the dynamic characteristics of the parallel grinding robot. To this end, they utilise digital simulation environments and tools for describing the kinematics and dynamics of mechanisms. In [8], the authors propose a hybrid control strategy for grinding and polishing robots based on adaptive impedance control. The results of simulations and experiments conducted by the authors indicate that the contact force is stable at 10 ± 3 N. In the article [9], the authors present the design of a novel expert system for robotic manipulators performing sanding tasks. They base their findings on experiments, using the UR5e robotic arm. The proposed strategy was based on the correlation found between vibrations and surface roughness. In article [10], the authors consider the force mode from the perspective of control algorithms in peg-in-hole assembly tasks. In another paper [11], the authors consider the use of a cobot in a robotic shoe polishing application for the shoe industry. In their research, they mainly focus on controlling the contact force of the tool, and toe shoe polishing is performed, achieving a good quality standard. In the paper [12], the authors introduce the relevant concepts of force control of grinding and polishing robots. It describes two modes of robot force control and then elaborates on the development of intelligent algorithms of hybrid force–position control and impedance control. In article [13], a novel high-bandwidth end-effector with active force control for robotic polishing is proposed. In [14], the authors propose safe and accurate force control in cooperative work with humans, through a series of comparative experiments with different contact forces and feed rates. The paper [15] describes a grinding system solution that uses force modes and motion path programming techniques in conjunction with Learning by Demonstration (LbD) techniques. In [16], a new method of impedance sliding mode control with adaptive fuzzy compensation according to Lyapunov’s theory is described. In papers [17,18,19], the conditions for the use of robots in robotic grinding operations are considered, using various technological parameters, e.g., frictional forces or vibration. Research presented in [20,21,22,23,24,25] indicates the considerable difficulty in achieving accurate and stable levels of force applied under force control modes, especially with low-levels of desired force and torque due to, among other reasons, sensor inaccuracies, control loop limitations, and inaccurate dynamic modelling of robot mechanisms.

Considering the literature review presented above, it should be noted that it is difficult to find sources from which integrators of automated systems with cobots could draw knowledge about the tuning of the force modes of the UR5e cobot. The research results presented in this article aim to reduce the scientific gap regarding the application of UR5e in low-force modes (in the range from 1 N to 10 N).

This article was written in response to the need to test the stability of the force mode, in the low-force range from 1 N to 10 N, in various scenarios of its operation in the workspace, with different definitions of force parameters and the controller regulating the force interaction of the robot’s end-effector, where the user can set the following parameters: Gain (understood as the stiffness of the dynamic system) and Damping. The findings of such research could be of significant value to cobot integrators seeking to implement these principles within their own automated production system projects, particularly those concerned with low-force industrial applications such as polishing or precision robotic grinding.

This article is organized as follows: Section 1 introduces the reader to the subject of cobot use and justifies the need for research and knowledge acquisition regarding the control of the UR5e cobot in low-force modes. Section 2 will describe the hardware and software components of the test bench and define the methodologies used in the experiments to determine the actual force values generated by the UR5e robot. The authors will undertake a review of scientific papers on robotic grinding and polishing using force modes. Section 3 will present the results obtained from the experiments in the form of tables and graphs. In Section 4, the results obtained will be discussed, and recommendations will be formulated. Section 5 will summarise the key results of this work and delineate the directions and scope of future scientific work on the research topic.

## 2. Materials and Methods

In this section, we describe the methods and materials used in the current work. Section 2.1 discuss the availability of the force mode parameters used in the UR5e robot software NI LabView 2021, which shape the ramifications of the control variables used in the following tests. We next describe the configuration of our experimental setup in Section 2.2.

The research in the current paper is concentrated around three experiments, described in subsequent sections:

- Experiment 1, described in Section 2.3, was performed to test the dependency of the effective level of the applied force on the position of the task in the workspace of the robot. The position was varied in two dimensions (X and Z axes), and the force was applied vertically downwards.
- Experiment 2, described in Section 2.4, was performed to establish the dependence of the effective level of the applied force on the desired level of the applied force and the distance from the base of the robot. In this scenario, the different levels of force (1–10 N) were applied in the horizontal direction.
- Experiment 3, described in Section 2.5, was designed to evaluate the dependence of the effective force parameters, such as the mean value, the overshoot, etc., on the force mode parameters set in the robot control software (e.g., Gain and Damping). In this experiment, the desired level of the force, the approach speed, the Gain, and the Damping are varied in a single location in the workspace of the robot.

In each of the experiments mentioned above, each data point was established with 12 repetitions of the force application. In each repetition, the end-effector was moving towards the contact point with the pre-set speed and force parameters, and the constant desired force was applied for the predefined duration. The contact segments were later identified based on (1) the digital output set in the control script upon contact and (2) reaching the force threshold of f_THR_ = 0.8 f_desired_. The objective metrics were computed based on the raw signal from the F/T sensor divided into individual contact segments and normalized with an offset according to the ambient level of the force in between the contact segments.

### 2.1. Availability of Force Mode Definition Options in the UR5e Robot User Interface

The UR5e user interface proposed by Universal Robots allows force modes to be programmed for a selected axis of robot operation. The interaction of forces may have an effect on a specific single axis of the robot, or alternatively on a few axes that are utilised during the assumed movement of the end-effector. According to the information provided in the UR5e cobot User Manual [26], force mode is intended for applications where the actual position of the Tool Centre Point (TCP) along a specific axis is not important, but instead the desired force along that axis is required. In the graphical user interface, the user has a choice of four force modes: Simple, Frame, Point, and Motion, in which the value can be set between 0 and 50 N [27]. In the Simple mode, a force value can only be specified for one axis. The user can select the option to express the force in either the Base or Tool frames. In Frame mode, it is similarly possible to specify a force interaction value in relation to Base or Tool frames. In each axis (degree of freedom), the value of the force can be defined in Newtons [N]. In addition, the Speed Limit [mm/s] parameter can be set. In Point mode, the task frame has the *y*-axis pointing from the robot TCP towards the origin of the selected feature. In Motion mode, the task frame will change with the direction of TCP movement. Here, the parameter values are set by the user, analogously to the previous cases.

In a more advanced form of force-mode programming, the user can additionally set Gain (Stiffness) and Damping values. It is acknowledged that these values may vary accordingly: Gain in a range from 0.0 to 2.0 (Universal Robots documentation recommends that a value larger than 1 can make force mode unstable) and Damping in a range from 0.0 (0.0 is no Damping) to 1.0 (1.0 is a full Damping) [27]. However, Universal Robots does not specify in the documentation how the indicated parameters should be set by the user to build their own integration applications, e.g., with materials of varying degrees of hardness, smoothness, and surface elasticity, or how they should be coupled with compliance devices and respond to the secondary impact of active end-effectors, e.g., rotary grinders.

In our work, we have used the script interface of the robot, where we have explicitly indicated the active and compliant axes, as well as the desired force level, approach speed, Gain and Damping, respectively, for each of the experimental scenarios.

### 2.2. Description of the Laboratory Bench Components Used in the UR5e Robot Force-Mode Tests

**Figure 1:**
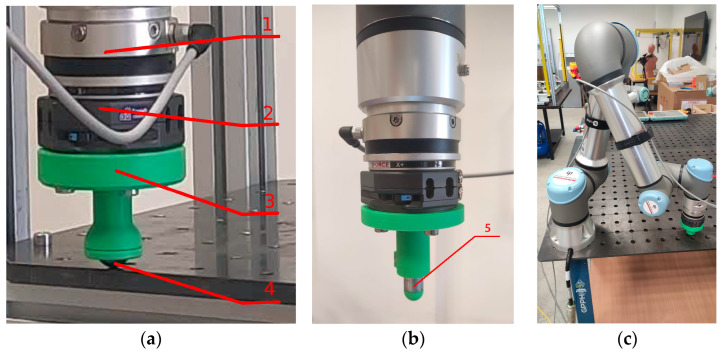
View of the main components of the laboratory stand; (**a**) view of the end-effector coupled to the UR5e cobot used in the measurement system in the second scenario; (1) OptoForce Hex six-axis force/torque sensor; (2) Wingman cobot tool changer; (3) 3D-printed custom end-effector; (4) free-rotating steel ball; (**b**) view of the end-effector coupled to UR5e used in the measurement system in the first scenario; (5) interchangeable end-effector tip with varied lengths; (**c**) view of the end-effector connected to the UR5e arm during the recording the measures data.

A laboratory test station was created for the purpose of conducting the planned research on the low-force modes of the UR5e cobot. The primary hardware element is the UR5e cobot arm, affixed to a bespoke welding and assembly table from company GPPH, with tabletop dimensions of 1200 × 800 mm (Figure 1).

The welding table provides proper support and stability for the robot and other hardware components used in the tests. A six-axis Hex force/torque sensor manufactured by OptoForce was attached to the robot’s wrist [28]. This sensor was utilised to measure the force exerted by the robot’s end-effector. According to the specifications, the typical noise level of the Hex sensor signal is 0.15 N, and its maximum sampling frequency is 500 Hz [28].

For the purpose of experiments involving the application of static force in different directions, a simple end-effector was designed in SolidWorks 2025 and prototyped using 3D printing technology, ending with a spherical, freely rotating steel ball. The rotating ball allows for reduced friction between the effector and the work surface and provides a precise single point of contact for the applied force. The indicated end-effector components are connected by the Wingman Cobot Tool Changer system with a standard ISO flange WM1-A-02-02-02 TripleA-robotics company (Figure 1a). The mass of all components of the solution attached to the robot flange was 580 g. The position of the TCP point in the robot’s parameter definition has been shifted by 116 mm from the robot’s default TCP point. All measurements were conducted at a constant ambient temperature of 22 °C under laboratory conditions.

In Experiment 1, where an analysis of the influence of the position of the force application point is considered, we have used a set of interchangeable end-effector tips (see Figure 1b) of different lengths (0.05 m, 0.1 m, 0.15 m, 0.20 m, 0.25 m, 0.30 m, 0.35 m, 0.40 m, 0.45 m, and 0.50 m) to facilitate the vertical offset of the contact point.

**Figure 2:**
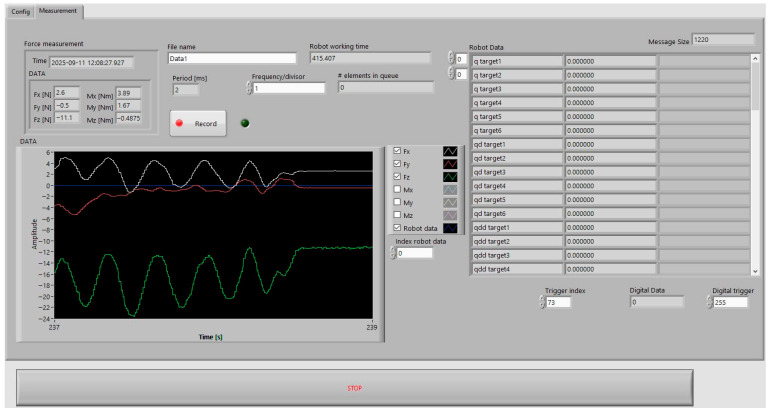
The main window of the user interface of the LabVIEW software (NI LabView 2021).

The software part of the test research station consists of a proprietary application developed in the LabVIEW environment, which allows for the recording, presentation, and saving of the used force/torque parameters from the OptoForce Hex sensor, as well as all current parameters available from the UR5e robot controller (Figure 2). Thus, there is no need to be directly connected by a cable to the robot and the additional sensor attached to its wrist. The program allows the user to define the location of data storage on the computer, in addition to manually resetting the force/torque sensor readings before and after each measurement. This process ensures the attainment of consistent measurement results.

The software is distinguished by an additional feature that facilitates the online monitoring of selected parameters in individual sensor measurement axes. These parameters are presented in graphical form, obviating the necessity for their prior storage. The program allows for the unique file names to be assigned and time tags to be automatically added to the file names to distinguish between recorded and stored measurement results. The application also allows the user to set the sampling frequency of the measured and recorded results.

### 2.3. Testing the Low-Force Mode of the UR5e Robot in Planes Parallel to the Mounting Surface of the Robot Base

**Figure 3:**
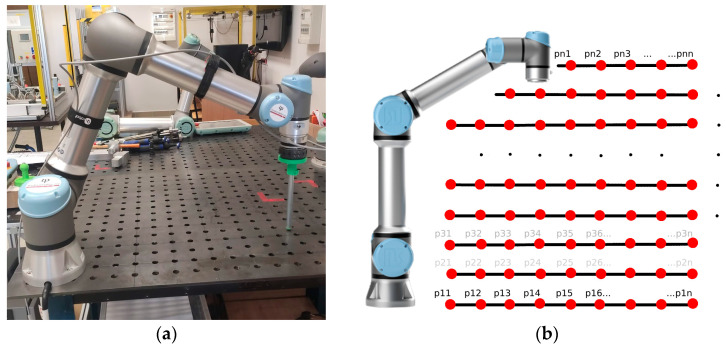
(**a**) View of the measurement station configuration for the UR5e robot, arranged for a scenario where measurements are performed in planes parallel to the robot base’s mounting surface with an end-effector with a removable pipe, with a length of 0.2 m; (**b**) schematic diagram of the measurement methodology for the assumed scenario.

To determine the UR5e robot arm’s ability to reproduce the set force value accurately within the range of 1 N to 10 N, an additional end-effector was constructed for the test stand. This structure was made using commercially available steel pipe and 3D printing technology (Figure 1b and Figure 3a). Thanks to the configuration of the test stand, it was possible to simulate the impact force of the end-effector on the surface at a specified value. In this case, the force was directed perpendicular to the mounting surface, in accordance with the *Z*-axis direction of the robot’s base coordinate system.

In a first measurement scenario, the UR5e robot arm performed the following actions in a specified position in the workspace: by moving it from the point of the workspace boundary closest to the *Z*-axis of the robot’s global coordinate system, linearly towards the workspace boundary, until the point where the robot could not perform the task due to reaching a singular position. It was assumed in the tests that the speed limit was 0.01 m/s and that the desired force in the *Z*-axis was 10 N. The height of the surface on which the end-effector acted was varied from 0.00 m (the robot base’s mounting surface) to 0.5 m, in 0.05 m intervals. At each measurement point, the robot performed a force interaction. This ultimately enabled 12 measurements to be recorded at each point in the working space at a given force value (Figure 3b). The measurements were limited to the working space above the robot base plane. The configuration used in the UR5e cobot arm tests is shown in Figure 3a.

The above-described measurement procedure was repeated at each subsequent measurement point for each successive upper layer until points were reached that could not be physically achieved due to the geometric limitations of the stand components.

In this experiment, we have used the default force-mode parameters, i.e., Gain = 1.0 and Damping = 0.005.

### 2.4. Force-Mode Testing of the UR5e Robot in Planes Perpendicular to the Mounting Surface of the Robot Base

**Figure 4:**
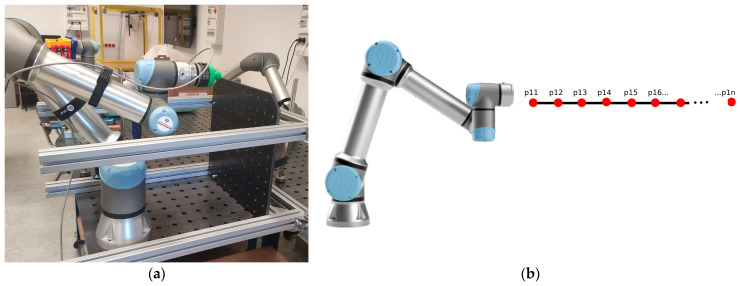
(**a**) Configuration of the UR5e robot measurement station for perpendicular measurement planes; (**b**) schematic diagram of the measurement methodology for the assumed scenario.

In the assumed measurement scenario, tests were performed with the bench configuration shown in Figure 4a. In this case, the steel top plate of the additional structure was positioned perpendicular to the base of the UR5e robot arm. This allowed the robot to exert a force ranging from 1 N to 10 N on the top plate, with its end-effector in the *X*-axis direction relative to the robot base. This was realized under the assumption of a speed limit of 0.001 m/s. The steel plate in the first measurement was set at a distance of 0.55 m, relative to the robot. Further measurements were taken at the following slab distances: 0.61 m, 0.65 m, 0.70 m, 0.75 m, 0.80 m, 0.85 m, 0.90 m, and 0.95 m. At one measurement point, the robot performed 12 repetitions of the end-effector interaction, with a set low force (Figure 4b).

In this experiment, we have used the default force-mode parameters, i.e., Gain = 1.0 and Damping = 0.005.

### 2.5. Testing the Force Mode of the UR5e Robot in a Fixed Position, Assuming Changes in the Robot Regulator Parameters over the Full Range of Values

In the advanced control mode of the force mode of the UR5e robotic arm, the operator can set the parameters of the impedance controller: Gain and Damping [27]. In the assumed measurement scenario, the end-effector of the UR5e robot acted forcibly on the surface of the welding table on which the robot arm was mounted (Figure 1b). Measurements were made under different force-mode impact scenarios, with assumed parameter changes. Table 1 shows the assumed variables in the UR5e force-mode measurements. Variations of these parameters over the full range of values made it possible to record 11,000 measurement results.

## 3. Results

This section presents the results from experiments conducted with the UR5e robot in low-force modes. Before the measurement of each set, a procedure was implemented to calibrate the force sensor affixed to the UR5e end-effector. All received results will be discussed. All figures illustrating the recorded measurement data from the experiments conducted have been archived in the repository at the following link (https://gitlab.com/kair_robotics/pomiary_sila, accessed on 25 January 2026). Measurement data from all the measurement scenarios are available by contacting this article’s corresponding author.

### 3.1. Results of Low-Force-Mode Tests in Planes Parallel to the Mounting Surface of the Robot Base (Experiment 1)

**Figure 5:**
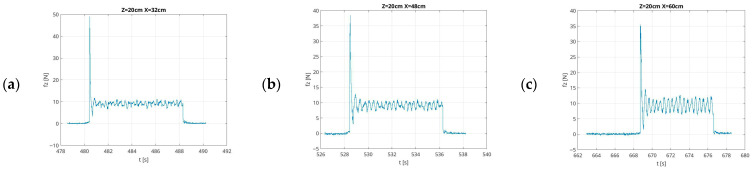
Example graphs of force measurement results in time, in low-force mode, with a 10 N setpoint, taken at X-distances of (**a**) 0.32 m, (**b**) 0.48 m, and (**c**) 0.60 m from the UR5e robot base, at a height of Z = 0.20 m.

Examples of recorded force values generated by the UR5e robot in low-force mode in the first scenario are shown in Figure 5. The figures illustrate the change in the amplitude generated by the UR5e robot force over time. The figures are presented at a set force value of f_z_ = 10 N, directed in the direction of gravity, at measurement distances from the robot base of X = 0.32 m, X = 0.48 m, and X = 0.60 m, at an interaction height Z = 0.20 m.

The measurement data were processed in the mathematical environment MATLAB R2025a to generate graphs of the X–Z plane of the UR5e robot’s workspace, highlighting areas of change in the force-mode setpoint. Table A1 (attached in the Appendix A section of the article) summarizes the coordinates of the measurement points used in the first measurement scenario (Z-coordinate and X-coordinate). The resulting data are presented through several key metrics, including the X, Z positions, the mean forces with the standard deviation, the mean max with the standard deviation, the root mean square error (RMSE) with the standard deviation, the mean absolute error (MAE) with the standard deviation, the mean rate with the standard deviation (ratio of the measured average force to the effective set force), and the mean overshoot with the standard deviation. Additionally, the mean proportion of time the force remained within ±10% (P10) and ±20% (P20) with the standard deviation of the setpoint is provided.

**Figure 6:**
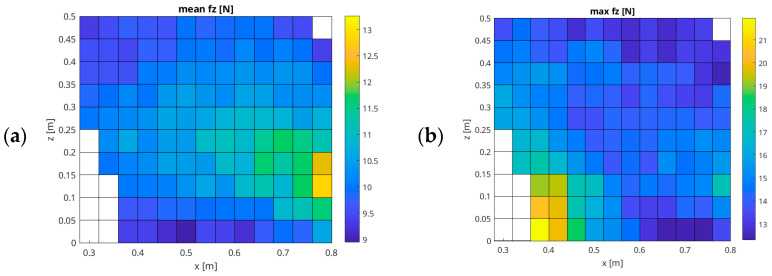

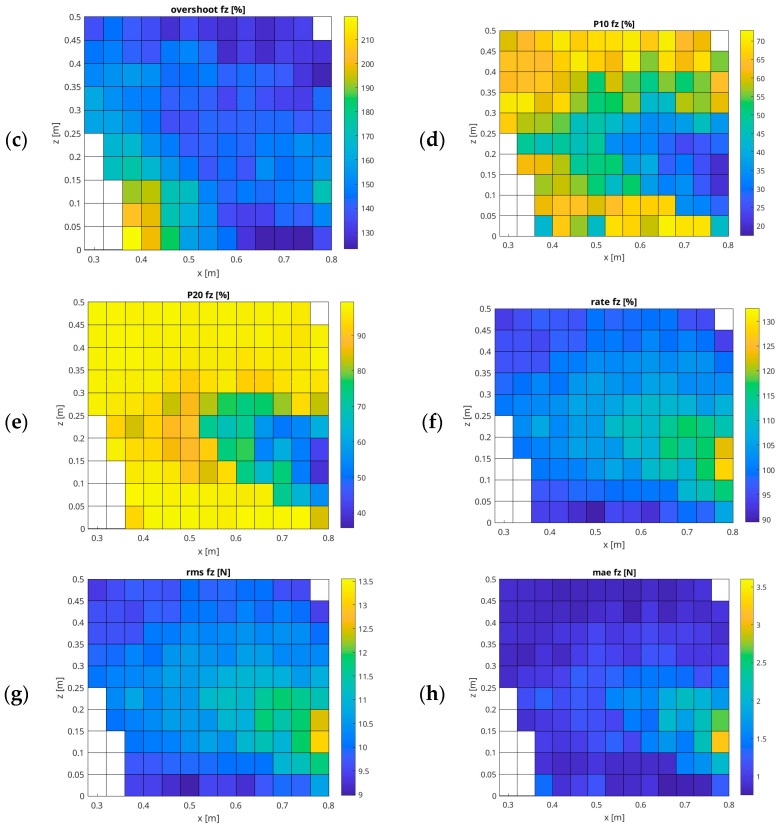
Results of force-mode testing in the X–Z plane, with an assumed force of 10 N and a speed of 0.01 m/s ((**a**) Mean f_z_ [N]; (**b**) Max f_z_ [N]; (**c**) Overshoot f_z_ [%]; (**d**) P10 f_z_ [%]; (**e**) P20 f_z_ [%]; (**f**) Rate f_z_ [%]; (**g**) RMSE f_z_ [N]; (**h**) MAE f_z_ [N]).

As illustrated in Figure 6, the sample family of graphs provides a comprehensive overview of the experimental data. The graphs include the following: a graph of the mean recorded force in points of the X–Z plane of the UR5e robot’s working space; a graph of the mean force f_z_ [N]; a graph of the max force f_z_ [N]; a graph of the overshoot f_z_ [%]; a graph of the proportion of time for which the obtained force value is within ±10% of the set force, P10 f_z_ [%]; a graph of the proportion of time for which the obtained force value is within ±20% of the set force, P20 f_z_ [%]; a graph showing the ratio of the average force obtained to the set value, Rate f_z_ [%]; a graph the root mean squared error, RMSE f_z_ [N]; and a graph mean squared error, MSE f_z_ [N]. Some measurements summarized in Table A2 (attached in the Appendix A section of this article) do not contain recorded values, because, at these specific points in the workspace, the UR5e cobot was unable to generate force interaction and entered a locked state due to its configuration and the presence of kinematic singularities at the workspace boundary.

An analysis of the remaining results in Table A1 indicates that in low-force mode, with a setpoint of 10 N, the UR5e cobot generates an actual mean force ranging from 8.95 N to 13.26 N within the workspace plane. Consequently, the exerted force is lower than the setpoint by an average of approximately 11.1% (with a minimum of 8.9% and a maximum of 32%). The average standard deviation from the set value for this experiment is approximately 0.09 N. This phenomenon reveals a systematic undershooting of the cobot’s force controller relative to the 10 N target.

The maximum recorded force across all measurement points ranged from 12.3 N to 21.96 N, which gives an average value of 17.13 N. Such significantly different values were recorded in the working space where the end-effector of the UR5e robot was located in the area with the following coordinates: X from 0.28 m to 0.45 m and Z from 0 m to 0.15 m, as well as at the edge of the cobot’s workspace at coordinates of X at approximately 0.8 m and Z in the range from 0.1 m to 0.15 m.

An analysis of the variability in the RMSE f_z_ and MAE f_z_ parameters shows that error values increase when the cobot operates at maximum reach at heights between 0.1 m and 0.2 m above the base level.

To evaluate the control system’s ability to maintain the force within specified tolerance bands following initial contact, an analysis of the P10 f_z_ and P20 f_z_ metrics was conducted.

Within the reach X-range of 0.30 m to 0.70 m and at Z-heights between 0.05 m and 0.10 m, and at Z-heights above 0.35 m, the UR5e cobot generates a very rapid force impact, which may indicate stable and rapid attainment of the set value. In the case of the P20 f_z_, over 90% of the area is achieved in the area above a Z height of 0.35 m. The maximum mean P10 f_z_ was 72.90%, and the maximum mean P20 f_z_ was 99.41%.

**Figure 7:**
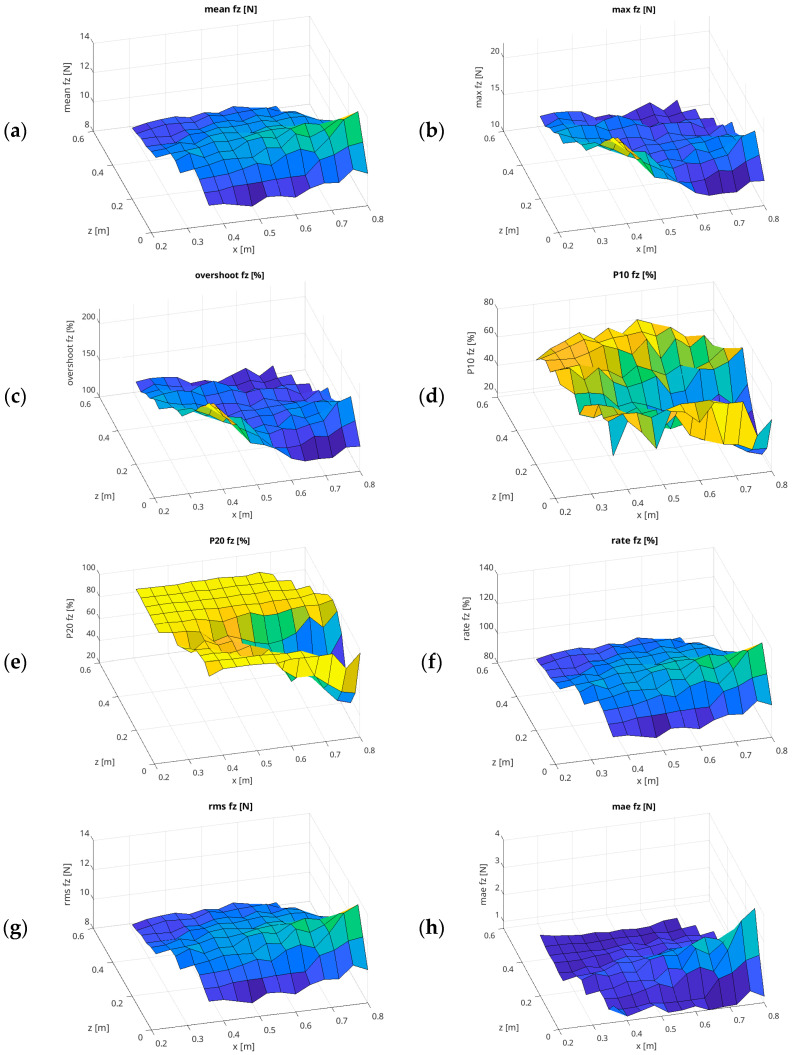
Results of force-mode testing in the X–Z plane, with an assumed force of 10 N and a speed of 0.01 m/s in surface representation ((**a**) Mean f_z_ [N]; (**b**) Max f_z_ [N]; (**c**) Overshoot f_z_ [%]; (**d**) P10 f_z_ [%]; (**e**) P20 f_z_ [%]; (**f**) Rate f_z_ [%]; (**g**) RMSE [N]; (**h**) MAE [N]).

Finally, the mean Rate f_z_ index was found to be 102.44%, with the best fit observed in the central part of the analysed workspace. As illustrated in Figure 7, a comparable array of graphs is presented, similar to that displayed in Figure 6, yet in a surface representation. This approach enables the reader to assess the changes in force values in the X–Z plane of the UR5e workspace in a more accessible manner, thereby complementing the presentation of the results from Figure 6.

Based on the measured values, we have created a polynomial regression model that estimates the influence of the position of the applied force on the X–Z plane on the achieved mean effective force. A 5th-order polynomial model has been found using the sklearn library, explaining the measured values with a coefficient of determination R^2^ = 0.9206. The model is presented in Equation (1).

(1) $$F_{est}=a_{50}x^{5}+a_{41}x^{4}z+a_{32}x^{3}z^{2}+a_{23}x^{2}z^{3}+a_{14}xz^{4}+a_{40}x^{4}+a_{31}x^{3}z+a_{22}x^{2}z^{2}+a_{13}xz^{3}+a_{04}z^{4}+a_{30}x^{3}\;+a_{21}x^{2}z+a_{12}xz^{2}+a_{03}z^{3}+a_{20}x^{2}+a_{11}xz+a_{02}z^{2}+a_{10}x+a_{01}z+a_{00}$$

where $F_{est}$ is the effective mean force estimated with regression [N], x is the horizontal distance [m], z is the vertical distance [m], and $a_{ij}$ represents the regression model coefficients:

a_50_ = −83.743693994, a_41_ = 757.66762544, a_32_ = 962.09708297, a_23_ = 1004.78951862, a_14_ = −1796.77088653, a_40_ = −50.75472445, a_31_ = −2215.77917356, a_22_ = −2191.61463043, a_13_ = 1007.65515765, a_04_ = 2080.11427655, a_30_ = 423.58980272, a_21_ = 2263.96271101, a_12_ = 695.80476164, a_03_ = −1198.4513006, a_20_ = −441.31116362, a_11_ = −868.63787017, a_02_ = 107.88350776, a_10_ = 173.21330683, a_01_ = 109.38006574, a_00_ = −14.218543639844997.

**Figure 8:**
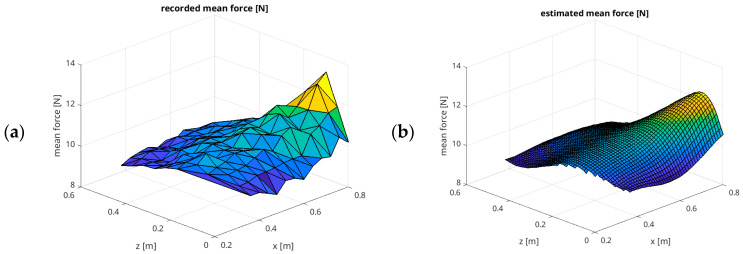
Results of regression modelling for experiment 1: mean force in the X–Z plane, with an assumed force of 10 N and a speed of 0.01 m/s in surface representation: (**a**) the measured mean force values; (**b**) the estimated mean force values.

Figure 8 compares the results from experiment 1, with the recorded mean force in the X–Z plane, with the results estimated by the polynomial model described by Equation (1).

### 3.2. Results of Low-Force-Mode Tests in Planes Perpendicular to the Mounting Surface of the Robot Base (Experiment 2)

**Figure 9:**
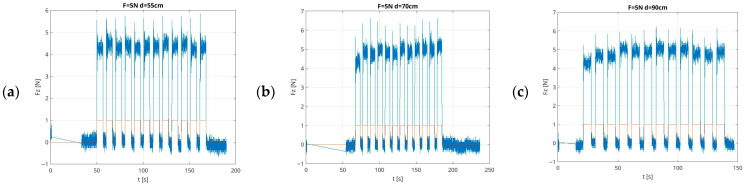
Example graphs from force measurement results in low-force mode, with a setpoint of 5 N, at distances of (**a**) 0.55 m, (**b**) 0.70 m, and (**c**) 0.95 m, relative to the UR5e robot base.

In experiment 2, 90 force measurements were recorded. Each single measurement set consisted of 12 repetitions for a defined force in the range of 1 N to 10 N. The end-effector of the UR5e robot applied force to the surface of a steel plate affixed to the welding table, perpendicular to the base of the UR5e robot arm mounting. The steel plate was displaced from the robot at predetermined distances. Measurements were taken in the range of 0.55 m to 0.95 m relative to the robot base while changing the force values in UR5e force mode. The recorded and processed results of the force measurements are summarised in Table A2 (attached in the Appendix A section of this article). The resulting data are presented with several key metrics, including the following: the specified force value; the distance from the robot base at which the UR5e robot’s end-effector exerted the specified force; the mean forces and their standard deviation; the mean maximum forces and its standard deviation; the mean root mean square error (RMSE) and its standard deviation; the mean absolute error (MAE) and its standard deviation; the mean rate (ratio of the measured average force to the effective set force) and its standard deviation; and the mean overshoot and its standard deviation. Additionally, the proportion of time that the force remained within ±10% (mean P10 f_x_) and ±20% (mean P20 f_x_) of the setpoint, with their respective standard deviations, are provided. It is worth noting that the P10 and P20 values for the low levels of force which approach the sensor accuracy level are only intended as an auxiliary performance metric. This summary allows the reader to quantify changes in the force generated by the UR5e cobot. Examples of sets of recorded measurements, before processing in Matlab R2025a, into summary, denoised graphs of changes in the measured quantity are shown in Figure 9.

**Figure 10:**
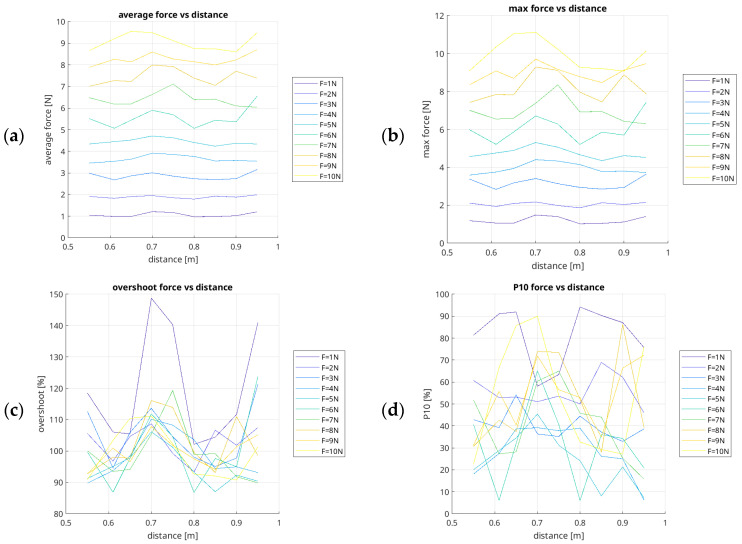

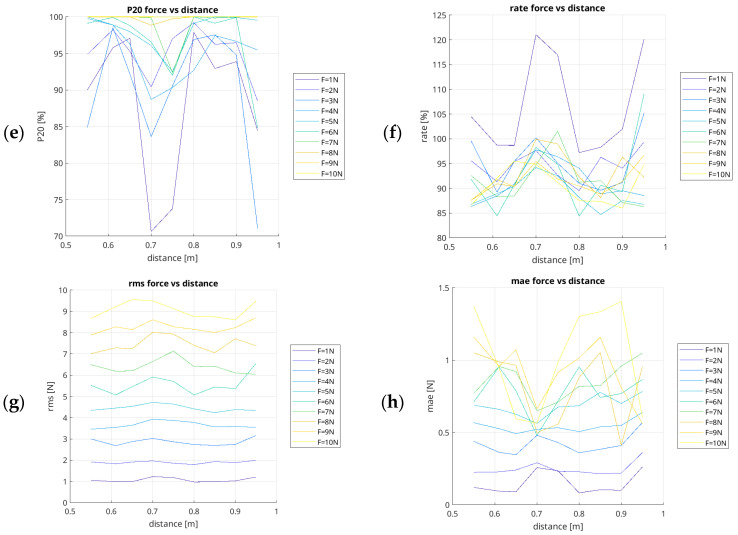
Diagrams of the relationship of the force parameters values related to distances (0.55 m, 0.61 m, 0.65 m, 0.70 m, 0.75 m, 0.80 m, 0.85 m, 0.90 m, and 0.95 m) of the end-effector in the *X*-axis of the UR5e robot in the low-force range, from 1 N to 10 N ((**a**) Average f_x_ [N]; (**b**) Max f_x_ [N]; (**c**) Overshoot f_x_ [%]; (**d**) P10 f_x_ [%]; (**e**) P20 f_x_ [%]; (**f**) Rate f_x_ [%]; (**g**) RMSE f_x_ [N]; (**h**) MAE f_x_ [N]).

The recorded measurement data was then used to compile summary results in the form of graphs showing the change in average force depending on the distance at which the set value was defined, ranging from 1 N to 10 N. The result is demonstrated in Figure 10. Each of the measures presented here refers to the change in force mode relative to the change in the end-effector’s distance of action on the *X*-axis, in the direction from the UR5e robot base.

An analysis of the experimental data reveals that the generated force values vary significantly as a function of the distance from the robot base. In proximity to the base (i.e., within the 0.55 m to 0.65 m range), the mean values of the measured force are generally lower than the setpoint commanded by the robot controller. For instance, with a set force of 5 N, the recorded mean force values were 4.34 N at a distance of 0.55 m, 4.44 N at 0.61 m, and 4.52 N at 0.65 m. Within this interval, the mean standard deviation from the setpoint is approximately 0.38 N.

At extended distances from the base (0.90 m to 0.95 m), the force values again deviate from the commanded setpoint. At a distance of 0.90 m, the mean measured force is typically lower than the requested values, whereas at 0.95 m, the value exceeds the setpoint (e.g., for a 7 N setpoint, the mean force was 6.09 N at 0.90 m, rising to 6.04 N at 0.95 m). The average standard deviation in this range is approximately 0.48 N.

Conversely, within the 0.75 m to 0.85 m range, the measured forces align most closely with the setpoints. This specific configuration proved to be the most effective for force tracking by the UR5e cobot’s controller. For a 9 N setpoint at distances of 0.70 m, 0.75 m, and 0.80 m, the mean recorded values were 8.59 N, 8.27 N, and 8.14 N, respectively, with an average deviation of approximately 0.44 N.

The results confirm that in the low-force range (1 N to 4 N), external disturbances, joint friction, and the specific kinematic configuration related to the end-effector’s distance from the workpiece constitute fundamental limitations to the reliable reproduction of the commanded force. In the 5 N to 7 N range, the measurements indicate more predictable system behaviour. The mean force accuracy improves as the distance from the base increases; specifically, in the 0.80 m to 0.95 m range, the mean measured values virtually correspond to the controller setpoints.

The cobot controller demonstrates its peak performance in the 8 N to 10 N range, where the force values are highly repeatable and accurately track the setpoints regardless of the distance. In this range of forces, the following was recorded: at a distance of 0.55 m from the robot base: mean P10 f_x_ = 28.14%, and mean P20 f_x_ = 100%; at 0.61 m: mean P10 f_x_ = 54.98%, and mean P20 f_x_ = 99.99%; at 0.65 m: mean P10 f_x_ = 54.51%, and mean P20 f_x_ = 100%; at 0.70 m: mean P10 f_x_ = 78.57%, and mean P20 f_x_ = 99.42%; at 0.75 m: mean P10 f_x_ = 61.29%, and mean P20 f_x_ = 99.89%; at 0.80 m: mean P10 fx = 45.92%, and mean P20 f_x_ = 100%; at 0.85 m: mean P10 f_x_ = 30.75%, and mean P20 f_x_ = f_x_ 100%; at 0.90 m: mean P10 f_x_ = 59.91%, and mean P20 f_x_ = 100%; at 0.95 m: mean P10 f_x_ = 62.52%, and mean P20 f_x_ = 99.99%. In the 8 N to 10 N range, P10 f_x_ and P20 f_x_ reach their maximum values, averaging 52.96% and 99.92%, respectively.

The P10 f_x_ and P20 f_x_ indices reach their lowest points in the low-force range (1 N to 4 N) and increase significantly in the medium-force range (5 N to 7 N). Low mean MAE values indicate excellent tracking performance (for example, at a distance of 0.70 m and force value of 8 N, the MAE value is 0.49 N; for a distance of 0.75 m and the same force value, the MAE value is 0.55 N).

A noteworthy finding is that at a distance of 0.75 m and 0.95 m, the force reproduction is characterized by the highest mean Std values across the entire 1 N to 10 N spectrum (respectively, mean of 0.54 N and 0.61 N).

The results confirm that force is generated most quickly when the robot’s end-effector is between 0.70 m and 0.75 m from the robot base and when it is in the area close to the boundary of the working space at the furthest points of the working space. The highest overshoot values are also visible at the boundaries of the tested working space.

Based on the measured values, we have created a polynomial regression model that estimates the relationship between the position of the applied force x and its value F on the achieved mean effective force. A 3rd-order polynomial model has been found using sklearn library, explaining the measured values with a coefficient of determination R^2^ = 0.9914. The model is presented in Equation (2).

(2) $$F_{est}=a_{30\;}F^{3}+a_{21}F^{2}x+a_{12}{Fx}^{2}+a_{03}x^{3}+a_{20}F_{x}+a_{02}x^{2}+a_{10}F+a_{00},$$

where F_est_ is the effective mean force estimated with regression [N], F is the desired force [N], x is the distance [m], and a_ij_ represents the regression model coefficients:

a_30_ = −2.25848950 × 10^−3^, a_21_ = −7.04412729 × 10^−3^, a_12_ = −7.05352720 × 10^−1^, a_03_ = 6.18279964 × 10^1^, a_20_ = 4.49583400 × 10^−2^, a_11_ = 1.15917071, a_02_ = −1.37368685 × 10^2^, a_10_ = 2.41803123 × 10^−1^, a_01_ = 9.98236625 × 10^1^, and a_00_ = −23.363085853841234

**Figure 11:**
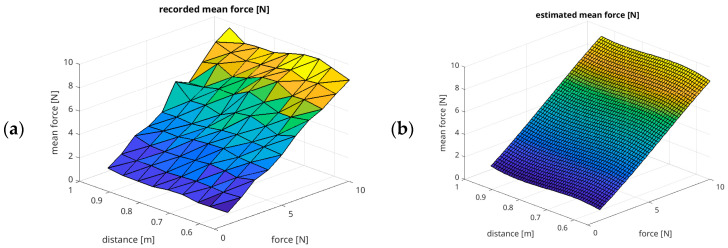
Results of regression modelling for experiment 2: mean force (from 1 N to 10 N) related to distances (0.55 m, 0.61 m, 0.65 m, 0.70 m, 0.75 m, 0.80 m, 0.85 m, 0.90 m, and 0.95 m) of the end-effector in *X*-axis: (**a**) the measured mean force values; (**b**) the estimated mean force values.

Figure 11 shows a comparison between the recorded mean force from experiment 2 and the estimated mean force using the polynomial model described by Equation (2).

### 3.3. Results of Low-Force-Mode Tests of the UR5e Robot in a Fixed Position, Assuming Changes in the Robot Controller Parameters (Experiment 3)

**Figure 12:**
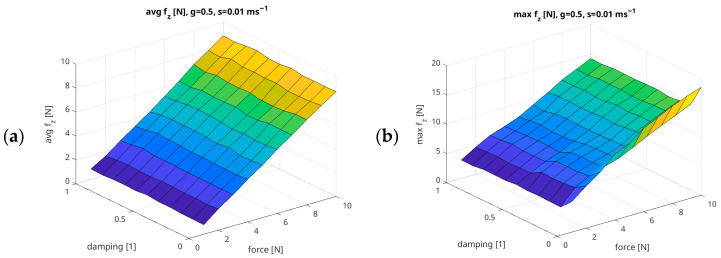

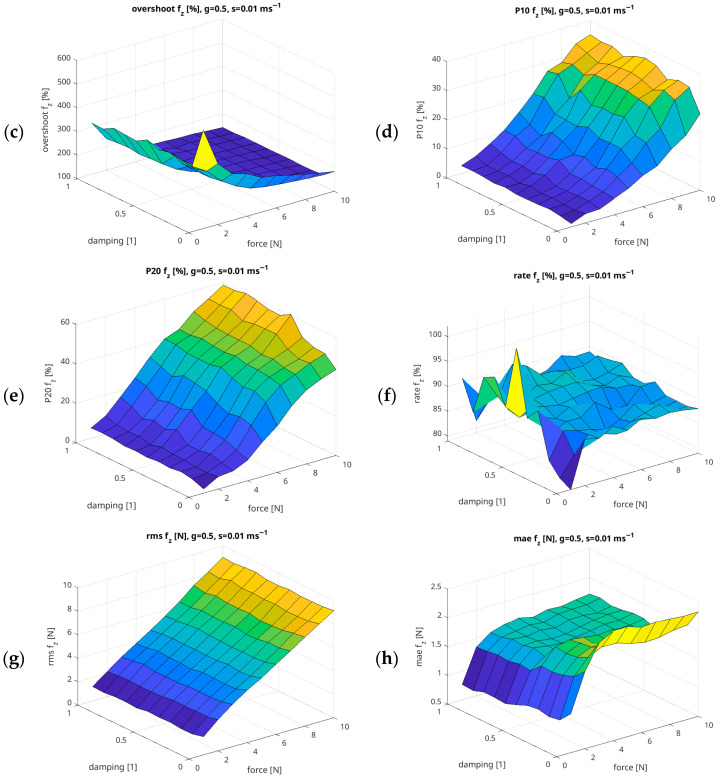
Example summary of force measurement results in relation to Force/Damping parameters, with fixed Gain = 0.5 and Speed = 0.01 m/s ((**a**) Avg f_z_ [N]; (**b**) Max f_z_ [N]; (**c**) Overshoot f_z_ [%]; (**d**) P10 f_z_ [%]; (**e**) P20 f_z_ [%]; (**f**) Rate f_z_ [%]; (**g**) RMSE f_z_ [N]; (**h**) MAE f_z_ [N]).

For this subsection, four groups of charts were developed based on the recorded measurement data. Figure 12 presents the relationships between the Damping parameter and the Force values generated by the UR5e robotic arm, assuming constant parameters of Gain = 0.5 and Speed = 0.01 m/s. Each group of charts provides a graphical representation of the following metrics: the mean value of the recorded force (Avg f_z_ [N]); the maximum value (Max f_z_ [N]); the force overshoot (Overshoot f_z_ [%]); the proportion of time for which the obtained force remains within ±10% of the setpoint (P10 f_z_ [%]); the proportion of time for which the obtained force remains within ±20% of the setpoint (P20 f_z_ [%]); the ratio of the mean obtained force to the commanded setpoint (Rate f_z_ [%]); the mean squared error (MSE f_z_ [N]); and the root mean squared error (RMSE f_z_ [N]).

In the force range from 6 N to 10 N, with damping values greater than 0.3, the lowest Overshoot f_z_ values are obtained, which may indicate that the UR5e robot can reproduce the set force value with high precision.

**Figure 13:**
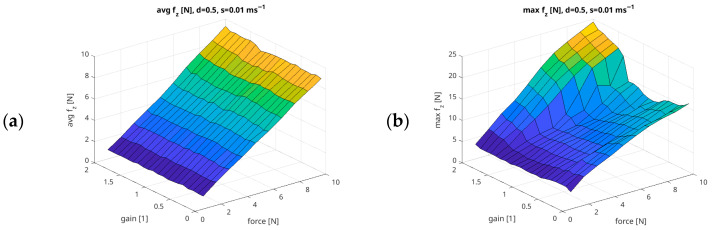

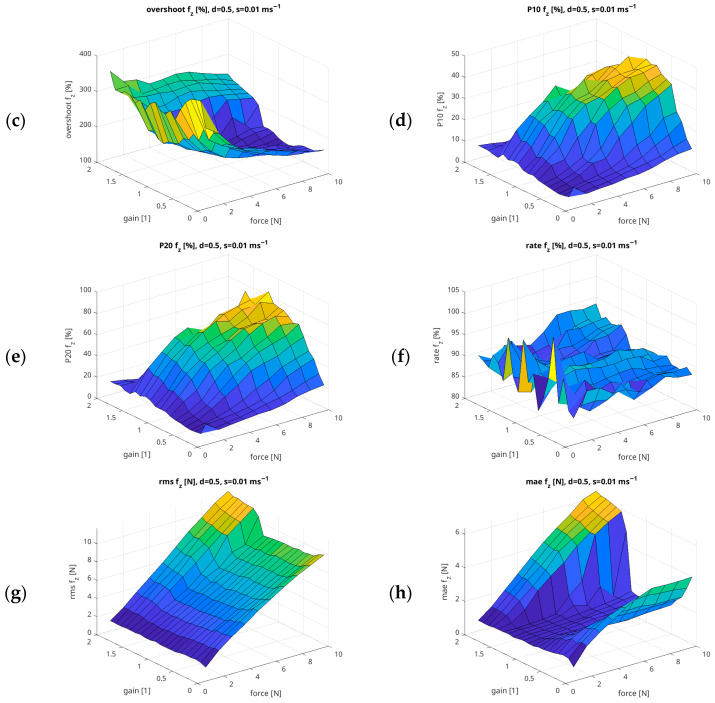
Example summary of force measurement results in relation to Force/Gain parameters, with fixed parameters of Damping = 0.5 and Speed = 0.01 m/s ((**a**) Avg f_z_ [N]; (**b**) Max f_z_ [N]; (**c**) Overshoot f_z_ [%]; (**d**) P10 f_z_ [%]; (**e**) P20 f_z_ [%]; (**f**) Rate f_z_ [%]; (**g**) RMSE f_z_ [N]; (**h**) MAE f_z_ [N]).

Figure 13 shows the relationships between the Gain parameter and the Force generated by the UR5e cobot arm, assuming a constant Damping parameter of 0.5 and Speed of 0.01 m/s.

**Figure 14:**
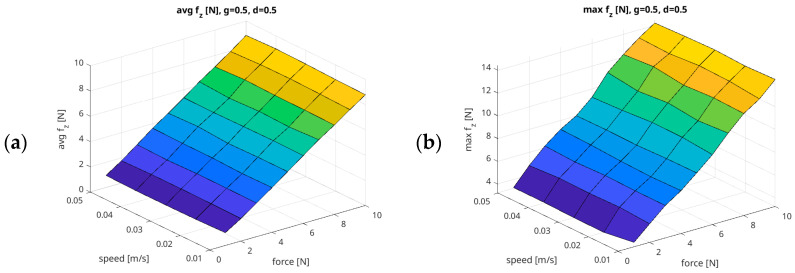

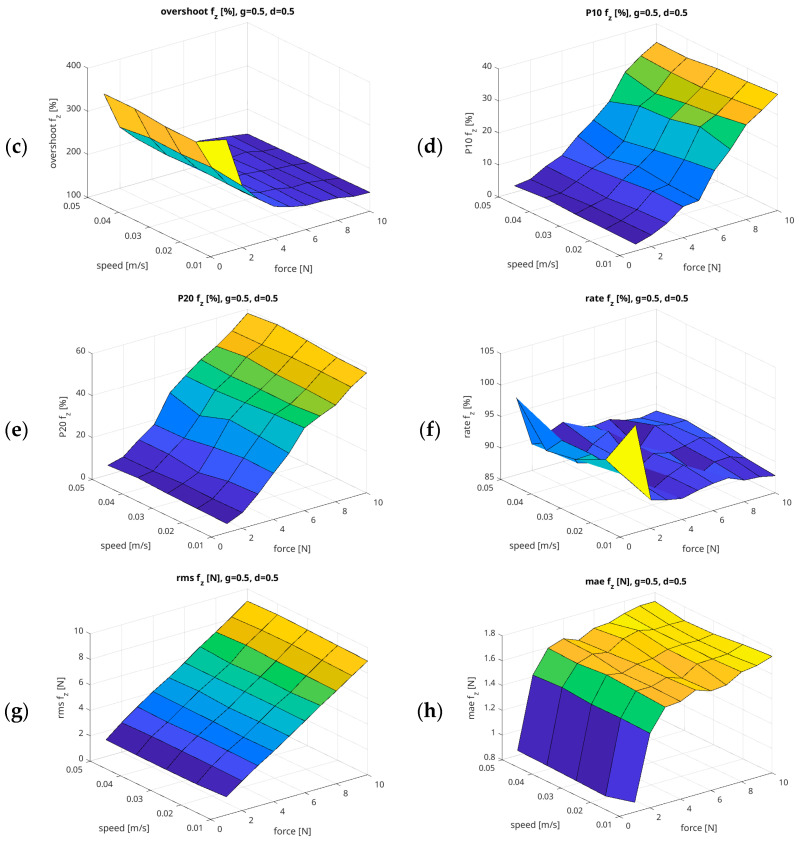
Example summary of force measurement results in relation to Force/Speed parameters, with fixed parameters of Gain = 0.5 and Damping = 0.5 ((**a**) Avg f_z_ [N]; (**b**) Max f_z_ [N]; (**c**) Overshoot f_z_ [%]; (**d**) P10 f_z_ [%]; (**e**) P20 f_z_ [%]; (**f**) Rate f_z_ [%]; (**g**) RMSE f_z_ [N]; (**h**) MAE f_z_ [N]).

Figure 14 shows the relationship between the Speed parameter and the Force generated by the UR5e robot arm, assuming a constant Damping = 0.5 and Gain = 0.5.

**Figure 15:**
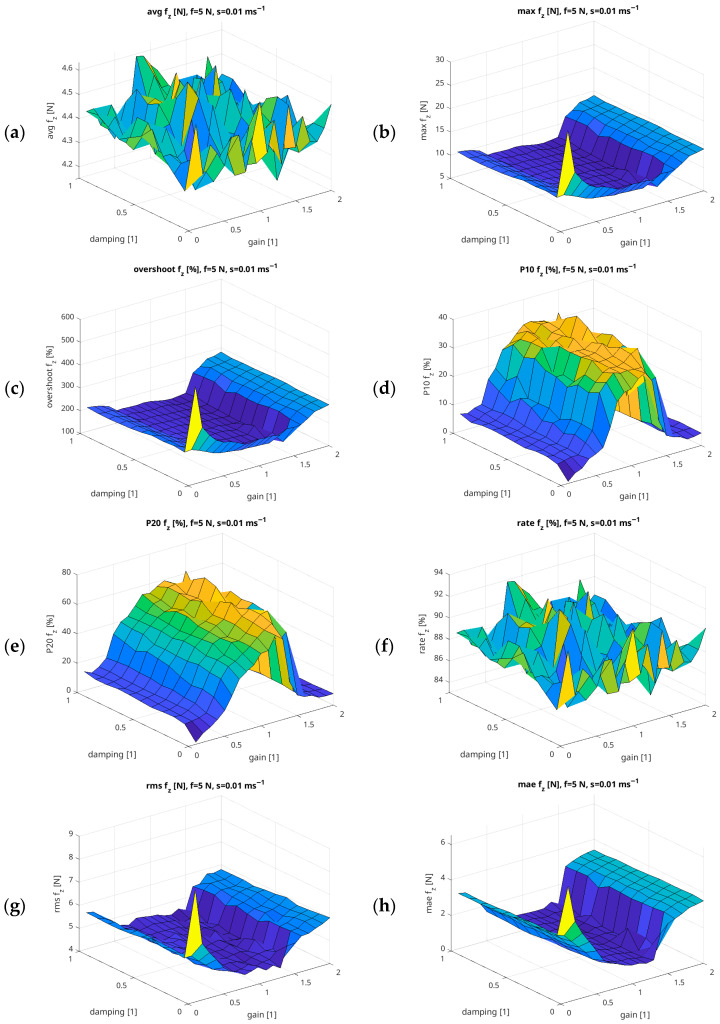
Example summary of force measurement results in relation to Gain/Damping parameters, with fixed Force = 5 N and Speed = 0.01 m/s ((**a**) Avg f_z_ [N]; (**b**) Max f_z_ [N]; (**c**) Overshoot f_z_ [%]; (**d**) P10 f_z_ [%]; (**e**) P20 f_z_ [%]; (**f**) Rate f_z_ [%]; (**g**) RMSE f_z_ [N]; (**h**) MAE f_z_ [N]).

Figure 15 shows the relationships between the Gain and Damping parameters, assuming a constant Force parameter of 2 N and Speed = 0.01 m/s. For each of the Figure 12, Figure 13, Figure 14 and Figure 15, a remaining group of graphs was generated with parameters analogous to those in Figure 12.

Due to the volume of the collected material, Figure 12, Figure 13, Figure 14 and Figure 15 are only representative examples of the obtained results. However, the entire research material served as a basis for formulating general conclusions about the force mode in the range from 1 N to 10 N for the UR5e cobot, taking into account the Gain, Damping, and Speed parameters that can be defined for the task by the system user. Due to the volume of 11,000 records, the results of the experiment in table form are included in the previously defined link in a repository rather than in the main text.

Based on the measured values, we created a polynomial regression model to estimate the relationship between the mean forces and the UR5e robot regulator parameters: Gain and Damping. The coefficient of determination for this model is R^2^ = 0.999. The model is presented in Equation (3).

(3) $$y=a_{0}+a_{1}g+a_{2}d+a_{3}f+a_{4}s+a_{5}g^{2}+a_{6}gd+a_{7}gf+a_{8}gs+a_{9}d^{2}+a_{10}df+a_{11}ds+a_{12}f^{2}+a_{13}fs\;+a_{14}s^{2}+a_{15}g^{3}+a_{16}g^{2}d+a_{17}g^{2}f+a_{18}g^{2}s+a_{19}gd^{2}+a_{20}gdf+a_{21}gds+a_{22}gf^{2}\;+a_{23}gfs+a_{24}gs^{2}+a_{25}d^{3}+a_{26}d^{2}f+a_{27}d^{2}s+a_{28}df^{2}+a_{29}dfs+a_{30}ds^{2}+a_{31}f^{3}\;+a_{32}f^{2}s+a_{33}fs^{2}+a_{34}s^{3},\;\;\;\;\;\;\;\;\;\;\;\;\;\;\;\;\;\;\;\;\;\;\;\;\;\;\;\;\;\;\;\;\;\;\;$$

where

a_0_ = −1.13 × 10^−2^, a_1_ = −4.96, a_2_ = −2.50 × 10^1^, a_3_ = 2.53, a_4_ = −1.01 × 10^1^, a_5_ = 1.58;

a_6_ = 1.04 × 10^1^, a_7_ = −2.25, a_8_ = 5.25, a_9_ = 3.75 × 10^1^, a_10_ = −5.95 × 10^−1^, a_11_ = 1.36 × 10^1^;

a_12_ = −7.13 × 10^−2^, a_13_ = 6.29 × 10^−1^, a_14_ = −2.33 × 10^2^, a_15_ = −2.61 × 10^−1^, a_16_ = −1.48;

a_17_ = 1.09, a_18_ = 2.13 × 10^1^, a_19_ = −6.60, a_20_ = 2.68 × 10^−1^, a_21_ = −1.03 × 10^1^;

a_22_ = 4.84 × 10^−2^, a_23_ = 4.37, a_24_ = −9.22 × 10^2^, a_25_ = −1.68 × 10^1^, a_26_ = 6.51 × 10^−2^;

a_27_ = −4.56, a_28_ = 1.42 × 10^−2^, a_29_ = 7.17 × 10^−1^, a_30_ = −4.15 × 10^1^, a_31_ = 3.67 × 10^−4^;

a_32_ = 1.65 × 10^−1^, a_33_ = −9.80 × 10^1^, a_34_ = 1.58 × 10^4^.

where g=gain, d=damping, f=force, s=speed.

**Figure 16:**
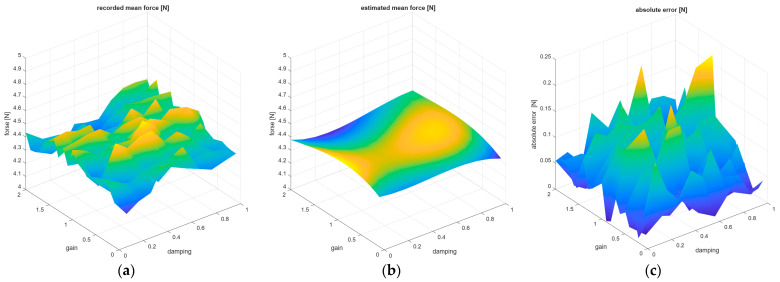
The relationship between the Gain/Damping UR5e regulator parameters and the following: (**a**) recorded mean forces and (**b**) estimated mean forces according to Equation (3). (**c**) Graphs showing the absolute error between the recorded and estimated mean forces, with fixed Force = 5 N and Speed = 0.05 m/s.

Figure 16 shows a comparison of the recorded mean forces value depending on the Gain and Damping parameters with the result of estimation of mean forces using the model from Equation (3). In addition, an analysis of absolute errors between the recorded mean values and those estimated by the model was performed, and the results of this error analysis is presented in Figure 16c.

**Figure 17:**
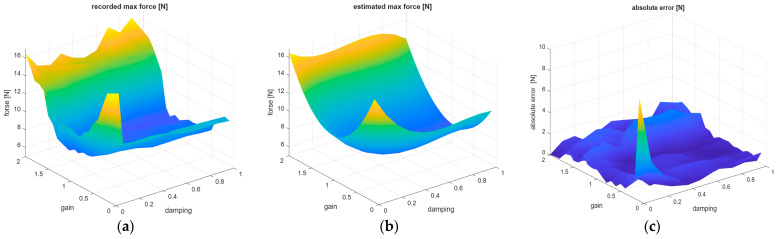
The relationship between the Gain/Damping UR5e regulator parameters and the following: (**a**) recorded max mean forces and (**b**) estimated max mean forces according to Equation (4). (**c**) Graphs showing the absolute error between the recorded and estimated mean max forces, with fixed Force = 5 N and Speed = 0.05 m/s.

Figure 17 shows a comparison of the recorded mean max forces value depending on the Gain and Damping parameters with the result of estimation of mean max forces using the model from Equation (4). In addition, an analysis of absolute errors between the recorded mean max values and those estimated by the model was performed, and the results of this absolute error analysis is presented in Figure 17c.

Additionally, a model was created to estimate the maximum value of mean forces of the UR5e robot and the regulator parameters, Gain and Damping, with a coefficient of determination R^2^ = 0.94. The model is presented in Equation (4).

(4) $$F=a_{0}+a_{1}g+a_{2}d+a_{3}f+a_{4}s+a_{5}g^{2}+a_{6}gd+a_{7}gf+a_{8}gs+a_{9}d^{2}+a_{10}df+a_{11}ds+a_{12}f^{2}+a_{13}fs\;+a_{14}s^{2}+a_{15}g^{3}+a_{16}g^{2}d+a_{17}g^{2}f+a_{18}g^{2}s+a_{19}gd^{2}+a_{20}gdf+a_{21}gds+a_{22}gf^{2}\;+a_{23}gfs+a_{24}gs^{2}+a_{25}d^{3}+a_{26}d^{2}f+a_{27}d^{2}s+a_{28}df^{2}+a_{29}dfs+a_{30}ds^{2}+a_{31}f^{3}\;+a_{32}f^{2}s+a_{33}fs^{2}+a_{34}s^{3},$$

where

a_0_ = 7.18, a_1_ = −4.96, a_2_ = −2.50 × 10^1^, a_3_ = 2.53, a_4_ = −1.01 × 10^1^, a_5_ = 1.58;

a_6_ = 1.04 × 10^1^, a_7_ = −2.25, a_8_ = 5.25 × 10^0^, a_9_ = 3.75 × 10^1^, a_10_ = −5.95 × 10^−1^, a_11_ = 1.36 × 10^1^;

a_12_ = −7.13 × 10^−2^, a_13_ = 0.63 × 10^0^, a_14_ = −2.33 × 10^2^, a_15_ = −0.26, a_16_ = −1.48;

a_17_ = 1.09, a_18_ = 2.13 × 10^1^, a_19_ = −6.60, a_20_ = 0.27, a_21_ = −1.03 × 10^1^, a_22_ = 0.05, a_23_ = 4.37 × 10^0^;a_24_ = −9.22 × 10^2^, a_25_ = −1.68 × 10^1^, a_26_ = 0.07, a_27_ = −4.56;

a_28_ = 0.01, a_29_ = 0.72, a_30_ = −4.15 × 10^1^, a_31_ = 0.0, a_32_ = 0.17, a_33_ = −9.80 × 10^1^, a_34_ = 1.58 × 10^4^.

The developed models show high consistency and are characterized by small force reproduction errors. Therefore, they can be used by future users in the development of practical low-force applications for the UR5e cobot.

## 4. Discussion

In this work, we have focused on experimental analysis of the performance of the force-control mode in the UR5e robot in the low-force regime. While we present the quantitative findings in the Section 3, this section is intended to provide a qualitative discussion of the obtained results and their synthesis into more general conclusions.

Despite the UR5e cobot’s high market popularity and its many fields of application, the authors were unable to find any scientific articles in bibliographic databases describing low-force-mode research focused on cobots. Therefore, the results of scientific work carried out for industry, which are described for classic industrial robots in references [29,30], were used as a reference. This allowed us to identify a number of problems related to control performance in low-force modes, especially in finishing processes involving complex shapes. The official documentation regarding force/torque sensor accuracy according to Universal Robots User Manuals UR5e is specified as 4 N [26], and this became the starting point for our own research. In [29], the authors note that there are certain drawbacks to commercial robotic manipulator control solutions, which usually become apparent at low interaction forces. In low-force modes, inertial forces—resulting, for example, from the movement of the tool along a curve—can become dominant [29]. The force sensor registers the sum of the interaction force and the inertial force, deceiving the control system. In extreme cases, the inertial force can exceed the contact force, causing destabilisation of the system and loss of contact between the tool and the machined surface. Work performed in [30] shows that tool contact forces below 2 N are typically not conducted using the force mode at all because the robot control system lacks the capability to control them effectively.

In experiment 1 we have analysed how the force projection metrics vary according to the location of the task in the workspace of the robot. The systematic undershooting of the cobot’s force controller relative to the 10 N target indicates a consistent deviation in low-force-mode operation. In precision machining applications, where the actual force must not exceed 10 N, the recorded overshot impacts may degrade machining quality or, in extreme cases, lead to workpiece damage due to an average dynamic overshoot of approximately 146.42%.

The fluctuations between maximum and minimum values indicate that the oscillations—particularly immediately after contact between the end-effector and the workpiece—may result from high gain in the force controller, which fails to decelerate the robotic arm sufficiently upon contact.

The increase in RMSE f_z_ and MAE f_z_ values at maximum reach and at heights between 0.1 m and 0.2 m above the base level is attributed to a loss of stiffness in the kinematic chain. Similarly, the UR5e force system loses precision in areas closest to the robot base and at maximum reach, which is associated with limited manipulability in these workspace zones.

In regions where the maximum mean P10 f_z_ and P20 f_z_ values were recorded, the cobot’s kinematics likely exhibit the highest structural stiffness. Reduced arm deflection translates into lower vibration amplitudes, allowing the controller to stabilize the force within the target intervals more rapidly. The best Rate f_z_ fit observed in the central part of the workspace further supports the conclusion that the middle workspace region provides the most favourable mechanical and control conditions for accurate force regulation.

In experiment 2, we have tested how the accuracy of the force mode depends on the reach of the robot arm and the desired level of the force. The observed deviations in force reproduction in proximity to the robot base (0.55 m to 0.65 m) can be attributed to the robot’s kinematic configuration and its proximity to workspace singularities.

Similarly, at extended distances from the base (0.90 m to 0.95 m), the deviation from the commanded setpoint indicates reduced stability of force tracking.

In the low-force range (1 N to 4 N), external disturbances, joint friction, and the specific kinematic configuration related to the end-effector’s distance from the workpiece constitute fundamental limitations to the reliable reproduction of the commanded force. The low values of P10 f_x_ and P20 f_x_ in this range reflect the inherent unpredictability of the system.

The improvement observed in the 5 N to 7 N range confirms enhanced predictability of the system. In the 8 N to 10 N range, robot posture changes have a negligible impact on force reproduction, indicating that the controller operates under the most favourable dynamic conditions in this interval.

At distances of 0.75 m and 0.95 m, the highest mean Std values across the entire force spectrum indicate reduced stability. Should a force-controlled task be required at these specific distances, a precision higher than ±0.57 N should not be expected. Any processes requiring finer control would necessitate additional mechanical damping at the end-effector.

The instability observed at a distance of approximately 0.75 m is most likely related to the controller implemented by Universal Robots, which, with default settings of Gain = 1.0 and Damping = 0.005 for the entire working space, when moving to the next point of the tested working space, generates a force that deviates significantly from the set value as a result of changes in the robot’s manipulability. This applies to each set value, but the phenomenon is most noticeable for low force values.

In experiments 1 and 2 it can be observed that the performance of the force control system degrades noticeably at the edges of the robot’s workspace in terms of accuracy of force projection. The highest overshoot values visible at the boundaries of the tested working space can also be attributed to the robot’s end-effector approaching singular positions where the robot has the least manipulability. Since the force projection index depends on the transpose of the manipulator Jacobian, it is expected that the force-mode accuracy will be dependent on the manipulability, and much lower as the robot arm approaches singular configurations, e.g., either the inner or the outer boundaries of the workspace. The manipulability is considered by the UR5e controller to an extent, as it can be noted that the force-mode operation is locked for the certain configurations of the arm (hence the N/A entries in results for experiments 1 and 2).

In experiment 3 we have performed a thorough investigation of the impact of the force-mode controller parameter tuning on the accuracy of the force representation. Analysing the results on the relationship between the Force and Damping parameters, it can be concluded that the P10 f_z_ and P20 f_z_ indicators decrease at low damping values. This may indicate that the UR5e force regulator system is oscillating, which is a negative phenomenon in force control. Setting higher force values improves the Rate f_z_ coefficient, indicating an increase in force-mode stability. It is difficult for the controller to achieve low setpoint values for the interaction force, which may be reflected in higher RSME f_z_ and MAE f_z_ values. Conversely, low RSME f_z_ and MAE f_z_ values are observed when higher forces are set. This can be interpreted as better mapping of the setpoint value within the higher force range. As the set force value increases beyond the 6 N threshold, the Rate f_z_ coefficient approaches 100%, suggesting that this is a threshold beyond which compensation for system nonlinearity is most effective.

It can be concluded that there is a relationship between the Force/Gain values, such that increasing the Gain value in the low force range (from 1 N to 4 N) results in high Overshoot f_z_ values, indicating that the set force is exceeded by more than 50%. However, this relationship is not entirely obvious within this range because, at these low values, the Rate f_z_ index improves. At high set force values close to 10 N, the Force/Gain relationship shows that high Gain does not significantly affect the reproduction of the set force value, as confirmed by the RSME f_z_ and MAE f_z_ values. The UR5e robot force controller operated most stably when the Gain values were between 0.3 and 0.6, with forces above the smallest analysed range, i.e., above 4 N.

Analysing the relationship between the Speed and Force parameters, it can be concluded that a low set Speed of around 0.01 m/s favours low Overshoot f_z_ values, which are associated with high precision in reproducing the set force value. However, Speed values above 0.01 m/s increase the RMSE f_z_ and MAE f_z_ values, suggesting a potential decrease in the UR5e cobot end-effector’s ability to accurately reproduce the set force. The robot exhibited the greatest stability when the set speed was below 0.04 m/s. For the 1 N mode, a minimum speed of 0.01 m/s is recommended. For a maximum force of 10 N within the tested range, the speed can be increased to 0.05 m/s without significantly compromising stability.

Considering the relationship between the Gain and Damping parameters, the analysed results clearly show that increasing the Gain parameter increases the speed at which the set force value is reached by the UR5e end-effector, while the damping parameter aims to reduce oscillations by dissipating force appropriately. At high Gain values and low Damping values, the system oscillates, which can be seen throughout the UR5e arm and during non-standard operation of the cobot drives. This is confirmed by the very high Std values. Increasing the Damping parameter with a constant gain value improves the P10 f_z_ and P20 f_z_ values, indicating “smoother and faster” reaching of the set force value without excessive oscillations. Conversely, low Gain values and high Damping values extend the time needed to reach the set force value, as can be seen by analysing the Rate f_z_ indicator, whose values fall below 80%. The most stable operating range was recorded for Gain values between 0.2 and 0.5 and Damping values between 0.3 and 0.6.

By analysing the results obtained in experiment 3, it is possible, as in the case of the previously presented analyses, to define conclusions for the force ranges from 1 N to 4 N, from 5 N to 7 N, and from 8 N to 10 N.

In the case of the first of the low-force ranges mentioned, 1–4 N, the results of the experiment show that the Gain and Damping controller parameters are most sensitive to change here. By increasing the robot controller Gain above 0.5, it can be observed that in the case of Overshoot f_z_, changes exceeding 200–300% occur. The P10 f_z_ and P20 f_z_ indicators are very low, as the robot spends most of its time oscillating after hitting the test plate. Damping is an important parameter here. At low values, the RSME f_z_ and MAE f_z_ error values are observed to be the highest. Therefore, for forces in the range of 1–4 N, it is recommended to use low Gain and high Damping values.

In the range of forces from 5 N to 7 N, it can be observed that an increase in the Gain value significantly improves the Rate f_z_ parameter, bringing it closer to 100%, which shortens the force rise time. When the Damping parameter is selected in the range from 0.1 to 0.3, the system stably achieves P20 f_z_ at 80%. It is therefore advisable to select a Gain of approximately 0.5 in this range. The robot force controller system then exhibits the best ratio between dynamics and precision in reproducing the setpoint value.

In the range of forces from 8 N to 10 N, high Gain values allow the robot’s internal resistance to be overcome and the set pressure force to be maintained. RMSE f_z_ errors are relatively the smallest compared to the set value. Then, the P10 f_z_ and P20 f_z_ indicators reach values of up to approx. 95%. In the case of the Damping parameter, its impact on force reproduction is smaller, as a higher force value stabilises the pressure of the end-effector.

Research has shown that the effectiveness of the low-force mode is highly non-linear. In the low force range (1–4 N), controller parameter selection must prioritize damping to compensate for sudden changes in kinetic energy upon contact with a rigid surface. In the 8 N to 10 N range, at higher loads, the UR5e control algorithm is highly resistant to geometric configuration changes, making it ideal for precise force tasks. Based on the obtained test results and considering the error criterion, the optimal settings of Gain, Damping, and Speed parameters were identified for each range of applied forces. The results are presented in Table 2.

It is worth noting that the standard deviation of the measured force for the desired force in the low range of measured values (1 N) is greatly influenced by and comparable to the signal noise (0.15 N) for measurement in the *Z*-axis using the HEX OptoForce force/torque sensor [28]. However, the standard deviation for forces in the measured range 2–10 N (see Table A1 in the Appendix A) exceeds the signal noise level and therefore are not fully explainable by the sensor uncertainty. For this reason, while we provide statistics on the full range of forces used in the experiment, we only consider the metrics generated for the forces in the range 2–10 N to be of meaningful value, while those for the 1 N force are attached in the interest of completeness and transparency.

We have performed regression modelling for the relationships investigated in the experiments described above. The regression models exhibit a good fit to the measured results. We hope that the proposed models may be used to draw recommendations to tune a range of force-mode parameters suited to execute specific low-force-mode tasks positioned at different areas in the workspace of the robot. Furthermore, more general recommendations at setting of the parameters expressed above may prove useful in force-mode application development.

## 5. Conclusions

The practical use of low-force modes is very important, among other things, in programming robotic operations for precision grinding and polishing of materials, in contour machining, in the assembly of delicate components, in testing and inspection at automated quality control stations, or when handling materials that require a delicate touch. Knowledge of the stability of the force mode in the X–Z plane of the UR5e cobot’s working space allows for increased accuracy in the planned application, as well as for the appropriate planning of a motion path generation strategy that will ensure accurate positioning in low-force mode.

In this work, we have presented results of experimental verification of the accuracy of the force mode of the UR5e robot in the regime of low levels of the desired force (1–10 N). We have performed a set of three experiments, in which we have tested the dependence of the parameters of the effective force (such as the mean forces and their standard deviation; the mean maximum forces and its standard deviation; the mean root mean square error (RMSE) and its standard deviation; the mean absolute error (MAE) and its standard deviation; the mean rate of force and its standard deviation; and the mean overshoot and its standard deviation) on the position of the task in the workspace of the robot, as well as the force controller parameters (desired level of the applied force, approach Speed, Gain, and Damping). We present the empirical results of these experiments, as well as polynomial models of the objective functions obtained through multivariate regression. Our results show that the performance of the UR5e force control is not uniform across its workspace. It is therefore advisable to optimize the prospective task location in order to achieve the desired level and stability of effective force. Furthermore, the Gain and Damping parameters of the controller can be tuned to achieve the desired dynamic characteristics of the applied force, such as the initial impact of the force.

The results of this study do not exhaust the research topic. Future research in this area will focus mainly on conducting low-force-mode tests in interaction with materials with different friction coefficients and Young’s moduli, using additional controlled compliance devices. This will allow for the full identification of force processes and the prototyping of our own robot controller, responsible for the precise generation of force in the range from 1 N to 10 N, which is a better alternative to the solution implemented by Universal Robots. The knowledge thus obtained will significantly improve the accuracy and repeatability of the UR5e robot’s positioning and allow the cobot to be consciously applied to new applications requiring the use of low-force modes, ranging from 1 N to 10 N. Future research will also be expanded to include additional analyses of low-force modes in various arm configurations, in workspaces also below the robot base, with the implementation of a proprietary force reproduction controller that will be tuned using artificial intelligence algorithms.

## Figures and Tables

**Table 1:** A set of parameters was defined for the investigation of the low-force interaction of the UR5e, covering the range from 1 N to 10 N.

Parameter	Minimal Value	Maximum Value
Gain (step 0.1)	0.1	2.0
Damping (step 0.1)	0.0	1.0
Speed (step 0.01 m/s)	0.01 m/s	0.05 m/s
Force (step 1 N)	1 N	10 N

**Table 2:** Selected Gain, Damping, and Speed parameters based on the error criterion.

Range of Applied Forces [N]	1–4	5–7	8–10
Gain	0.5	1.0	2.0
Damping	0.7	0.6	0.8
Speed [m/s]	0.05	0.03	0.01

## Data Availability

The data presented in this study are available on request from the corresponding author.

## Appendix A

**Table A1:** Summary of the results of the experiment carried out according to the first post-measurement scenario in the X–Z plane (set values: Force in *X*-axis = 10 N and Speed Limits = 0.01 m/s).

X [m]	Z [m]	Mean Force ± Std [N]	Mean Max ± Std [N]	Mean RMSE ± Std [N]	Mean MAE ± Std [N]	Mean Rate ± Std [%]	Mean Overshoot ± Std [%]	Mean P10 ± Std [%]	Mean P20 ± Std [%]
0.28	0	NA *							
0.32	0	NA *							
0.36	0	9.375 ± 1.162	21.958 ± 0.663	9.463 ± 1.225	1.371 ± 0.655	93.749 ± 11.623	219.583 ± 6.626	42.474 ± 10.271	93.303 ± 17.905
0.4	0	9.370 ± 0.067	19.917 ± 0.434	9.431 ± 0.068	0.911 ± 0.035	93.699 ± 0.673	199.167 ± 4.345	65.941 ± 3.865	98.564 ± 0.208
0.44	0	9.157 ± 0.128	18.550 ± 0.342	9.207 ± 0.131	1.035 ± 0.087	91.567 ± 1.280	185.500 ± 3.425	56.459 ± 7.306	98.581 ± 0.263
0.48	0	8.946 ± 0.068	15.808 ± 0.326	8.981 ± 0.070	1.188 ± 0.057	89.465 ± 0.676	158.083 ± 3.260	43.656 ± 5.613	98.538 ± 0.316
0.52	0	9.461 ± 0.072	15.275 ± 0.148	9.509 ± 0.074	0.854 ± 0.032	94.609 ± 0.723	152.750 ± 1.485	68.897 ± 2.375	97.966 ± 0.154
0.56	0	9.403 ± 0.063	14.458 ± 0.151	9.449 ± 0.065	0.869 ± 0.041	94.032 ± 0.635	144.583 ± 1.505	67.172 ± 2.706	96.975 ± 0.078
0.6	0	9.194 ± 0.107	12.958 ± 0.239	9.223 ± 0.109	0.949 ± 0.086	91.943 ± 1.072	129.583 ± 2.392	58.610 ± 6.547	98.879 ± 0.408
0.64	0	9.675 ± 0.094	12.367 ± 0.187	9.715 ± 0.097	0.753 ± 0.011	96.748 ± 0.943	123.667 ± 1.875	71.503 ± 1.590	98.573 ± 0.803
0.68	0	9.902 ± 0.046	12.417 ± 0.185	9.948 ± 0.048	0.792 ± 0.034	99.018 ± 0.463	124.167 ± 1.850	69.406 ± 3.492	98.400 ± 0.681
0.72	0	9.821 ± 0.047	12.300 ± 0.160	9.868 ± 0.049	0.810 ± 0.030	98.211 ± 0.467	123.000 ± 1.595	69.306 ± 2.895	98.865 ± 0.710
0.76	0	10.608 ± 0.081	13.492 ± 0.183	10.690 ± 0.083	1.242 ± 0.052	106.082 ± 0.805	134.917 ± 1.832	43.822 ± 2.471	84.025 ± 3.964
0.8	0	10.192 ± 0.076	13.375 ± 0.129	10.257 ± 0.079	0.986 ± 0.042	101.920 ± 0.758	133.750 ± 1.288	54.021 ± 3.083	96.250 ± 0.534
0.28	0.05	NA *							
0.32	0.05	NA *							
0.36	0.05	9.653 ± 0.062	20.492 ± 0.425	9.729 ± 0.063	0.920 ± 0.037	96.531 ± 0.618	204.917 ± 4.252	60.896 ± 3.406	98.674 ± 0.149
0.4	0.05	9.663 ± 0.082	19.817 ± 0.497	9.733 ± 0.083	0.860 ± 0.028	96.632 ± 0.816	198.167 ± 4.970	64.918 ± 2.190	98.498 ± 0.159
0.44	0.05	9.840 ± 0.097	17.342 ± 0.340	9.908 ± 0.105	0.877 ± 0.078	98.395 ± 0.969	173.417 ± 3.397	64.556 ± 5.089	97.872 ± 0.991
0.48	0.05	9.863 ± 0.031	16.367 ± 0.317	9.936 ± 0.032	0.953 ± 0.039	98.632 ± 0.306	163.667 ± 3.172	59.776 ± 2.102	97.753 ± 0.251
0.52	0.05	9.892 ± 0.060	15.300 ± 0.313	9.956 ± 0.064	0.882 ± 0.061	98.924 ± 0.597	153.000 ± 3.133	64.194 ± 4.415	97.760 ± 0.170
0.56	0.05	9.964 ± 0.078	13.550 ± 0.131	10.021 ± 0.081	0.866 ± 0.062	99.636 ± 0.777	135.500 ± 1.314	66.934 ± 4.770	97.036 ± 0.137
0.6	0.05	9.995 ± 0.116	13.250 ± 0.239	10.050 ± 0.125	0.865 ± 0.102	99.949 ± 1.162	132.500 ± 2.393	66.795 ± 8.370	96.462 ± 0.905
0.64	0.05	9.990 ± 0.079	13.350 ± 0.258	10.045 ± 0.083	0.864 ± 0.053	99.895 ± 0.792	133.500 ± 2.576	67.158 ± 4.647	97.222 ± 0.287
0.68	0.05	10.894 ± 0.081	14.333 ± 0.202	11.002 ± 0.082	1.519 ± 0.042	108.944 ± 0.812	143.333 ± 2.015	34.359 ± 1.907	72.922 ± 1.780
0.72	0.05	11.133 ± 0.049	14.025 ± 0.171	11.249 ± 0.052	1.675 ± 0.036	111.330 ± 0.494	140.250 ± 1.712	32.852 ± 1.413	63.410 ± 1.982
0.76	0.05	11.599 ± 0.101	15.108 ± 0.138	11.748 ± 0.103	2.059 ± 0.076	115.992 ± 1.006	151.083 ± 1.379	28.108 ± 1.013	55.147 ± 1.617
0.8	0.05	11.756 ± 0.179	15.700 ± 0.234	11.917 ± 0.190	2.197 ± 0.154	117.561 ± 1.786	157.000 ± 2.335	27.646 ± 1.991	52.241 ± 2.609
0.28	0.1	NA *							
0.32	0.1	NA *							
0.36	0.1	9.997 ± 0.083	19.192 ± 0.287	10.086 ± 0.086	1.012 ± 0.047	99.967 ± 0.827	191.917 ± 2.875	57.012 ± 2.401	96.401 ± 1.365
0.4	0.1	10.061 ± 0.074	19.367 ± 0.303	10.148 ± 0.076	0.962 ± 0.060	100.612 ± 0.742	193.667 ± 3.025	58.753 ± 3.120	97.159 ± 0.992
0.44	0.1	10.124 ± 0.085	17.275 ± 0.344	10.208 ± 0.088	1.012 ± 0.053	101.244 ± 0.847	172.750 ± 3.441	56.364 ± 2.543	95.785 ± 1.776
0.48	0.1	10.333 ± 0.088	16.975 ± 0.545	10.431 ± 0.092	1.146 ± 0.055	103.334 ± 0.879	169.750 ± 5.446	51.849 ± 2.813	90.604 ± 3.410
0.52	0.1	10.596 ± 0.106	14.967 ± 0.250	10.693 ± 0.114	1.282 ± 0.102	105.962 ± 1.060	149.667 ± 2.498	43.824 ± 4.123	84.317 ± 3.704
0.56	0.1	10.335 ± 0.083	14.242 ± 0.227	10.410 ± 0.091	1.077 ± 0.096	103.351 ± 0.829	142.417 ± 2.275	52.625 ± 5.471	94.125 ± 3.214
0.6	0.1	10.937 ± 0.081	14.717 ± 0.208	11.046 ± 0.086	1.517 ± 0.069	109.366 ± 0.805	147.167 ± 2.082	37.261 ± 1.499	73.924 ± 2.718
0.64	0.1	11.192 ± 0.135	14.350 ± 0.215	11.308 ± 0.149	1.696 ± 0.140	111.917 ± 1.349	143.500 ± 2.153	32.444 ± 2.481	65.167 ± 4.268
0.68	0.1	10.864 ± 0.031	14.800 ± 0.200	10.969 ± 0.031	1.482 ± 0.028	108.637 ± 0.312	148.000 ± 2.000	34.729 ± 1.260	74.832 ± 1.793
0.72	0.1	11.714 ± 0.100	15.067 ± 0.306	11.872 ± 0.108	2.182 ± 0.088	117.139 ± 1.002	150.667 ± 3.055	26.672 ± 1.247	52.154 ± 2.139
0.76	0.1	12.835 ± 0.089	17.258 ± 0.223	13.099 ± 0.096	3.219 ± 0.084	128.354 ± 0.891	172.583 ± 2.234	20.509 ± 1.265	39.602 ± 1.280
0.8	0.1	13.259 ± 0.242	17.833 ± 0.392	13.549 ± 0.255	3.606 ± 0.206	132.587 ± 2.420	178.333 ± 3.916	17.391 ± 0.726	35.699 ± 1.326
0.28	0.15	NA *							
0.32	0.15	9.970 ± 0.057	16.983 ± 0.351	10.042 ± 0.059	0.904 ± 0.056	99.699 ± 0.565	169.833 ± 3.512	61.446 ± 2.529	97.573 ± 0.692
0.36	0.15	10.141 ± 0.051	17.500 ± 0.374	10.224 ± 0.053	0.977 ± 0.027	101.406 ± 0.511	175.000 ± 3.742	60.821 ± 1.445	94.311 ± 1.162
0.4	0.15	10.274 ± 0.086	16.667 ± 0.368	10.355 ± 0.091	1.016 ± 0.068	102.738 ± 0.858	166.667 ± 3.676	57.812 ± 3.177	93.595 ± 2.667
0.44	0.15	10.519 ± 0.082	15.475 ± 0.171	10.609 ± 0.087	1.190 ± 0.070	105.189 ± 0.822	154.750 ± 1.712	48.989 ± 3.148	86.437 ± 2.051
0.48	0.15	10.478 ± 0.092	14.708 ± 0.247	10.561 ± 0.096	1.149 ± 0.068	104.776 ± 0.915	147.083 ± 2.466	50.828 ± 3.411	88.080 ± 2.566
0.52	0.15	10.412 ± 0.084	13.900 ± 0.148	10.486 ± 0.088	1.081 ± 0.065	104.120 ± 0.841	139.000 ± 1.477	52.084 ± 2.992	91.642 ± 3.081
0.56	0.15	10.949 ± 0.127	14.392 ± 0.173	11.050 ± 0.138	1.482 ± 0.128	109.489 ± 1.272	143.917 ± 1.730	37.168 ± 4.191	74.397 ± 4.354
0.6	0.15	10.814 ± 0.099	13.833 ± 0.150	10.906 ± 0.106	1.384 ± 0.088	108.139 ± 0.993	138.333 ± 1.497	39.748 ± 2.744	77.709 ± 3.365
0.64	0.15	11.656 ± 0.104	15.233 ± 0.150	11.799 ± 0.110	2.087 ± 0.091	116.561 ± 1.039	152.333 ± 1.497	27.503 ± 1.451	53.754 ± 2.794
0.68	0.15	11.305 ± 0.117	14.525 ± 0.196	11.427 ± 0.126	1.795 ± 0.107	113.052 ± 1.171	145.250 ± 1.960	31.132 ± 2.422	61.454 ± 3.740
0.72	0.15	11.690 ± 0.160	14.933 ± 0.284	11.848 ± 0.170	2.168 ± 0.138	116.904 ± 1.595	149.333 ± 2.839	25.320 ± 1.489	52.179 ± 2.935
0.76	0.15	12.297 ± 0.185	15.525 ± 0.398	12.472 ± 0.203	2.660 ± 0.175	122.968 ± 1.854	155.250 ± 3.980	20.094 ± 1.224	42.526 ± 1.791
0.8	0.15	11.667 ± 0.145	15.825 ± 0.270	11.803 ± 0.150	2.083 ± 0.124	116.672 ± 1.449	158.250 ± 2.701	25.962 ± 1.456	52.470 ± 3.482
0.28	0.2	NA *							
0.32	0.2	10.238 ± 0.052	16.592 ± 0.353	10.332 ± 0.055	1.135 ± 0.044	102.382 ± 0.521	165.917 ± 3.528	48.990 ± 3.031	92.521 ± 1.637
0.36	0.2	10.636 ± 0.039	16.692 ± 0.318	10.742 ± 0.041	1.313 ± 0.043	106.358 ± 0.392	166.917 ± 3.175	45.393 ± 2.695	83.866 ± 1.225
0.4	0.2	10.201 ± 0.072	15.392 ± 0.320	10.292 ± 0.075	1.138 ± 0.049	102.009 ± 0.721	153.917 ± 3.204	48.564 ± 2.808	93.046 ± 2.196
0.44	0.2	10.470 ± 0.060	14.783 ± 0.185	10.564 ± 0.060	1.230 ± 0.037	104.701 ± 0.595	147.833 ± 1.850	45.191 ± 2.117	87.507 ± 1.523
0.48	0.2	10.431 ± 0.060	13.992 ± 0.168	10.515 ± 0.062	1.166 ± 0.037	104.308 ± 0.601	139.917 ± 1.676	48.651 ± 1.845	89.717 ± 1.998
0.52	0.2	10.979 ± 0.058	14.150 ± 0.188	11.088 ± 0.060	1.553 ± 0.045	109.790 ± 0.579	141.500 ± 1.883	34.459 ± 2.190	72.625 ± 1.463
0.56	0.2	11.097 ± 0.087	14.408 ± 0.231	11.209 ± 0.091	1.638 ± 0.062	110.970 ± 0.872	144.083 ± 2.314	32.727 ± 1.793	67.854 ± 2.564
0.6	0.2	10.924 ± 0.118	14.258 ± 0.348	11.038 ± 0.126	1.571 ± 0.100	109.243 ± 1.183	142.583 ± 3.476	33.299 ± 1.803	72.211 ± 3.679
0.64	0.2	11.422 ± 0.094	14.817 ± 0.402	11.553 ± 0.100	1.907 ± 0.085	114.223 ± 0.937	148.167 ± 4.019	28.797 ± 1.518	56.901 ± 2.500
0.68	0.2	11.697 ± 0.112	14.783 ± 0.221	11.832 ± 0.120	2.127 ± 0.105	116.974 ± 1.120	147.833 ± 2.209	24.235 ± 1.448	51.258 ± 2.757
0.72	0.2	11.491 ± 0.118	15.308 ± 0.207	11.624 ± 0.125	1.980 ± 0.103	114.906 ± 1.176	153.083 ± 2.065	26.350 ± 1.632	54.366 ± 3.038
0.76	0.2	11.188 ± 0.070	15.100 ± 0.141	11.302 ± 0.074	1.721 ± 0.063	111.877 ± 0.699	151.000 ± 1.414	29.094 ± 1.284	62.071 ± 1.879
0.8	0.2	10.790 ± 0.124	14.925 ± 0.347	10.888 ± 0.129	1.403 ± 0.084	107.902 ± 1.242	149.250 ± 3.467	36.972 ± 2.184	82.712 ± 5.807
0.28	0.25	9.999 ± 0.106	15.892 ± 0.596	10.065 ± 0.110	0.869 ± 0.036	99.985 ± 1.062	158.917 ± 5.961	66.011 ± 2.908	97.520 ± 0.290
0.32	0.25	10.155 ± 0.060	15.850 ± 0.366	10.232 ± 0.066	0.965 ± 0.058	101.549 ± 0.602	158.500 ± 3.656	57.825 ± 2.911	96.009 ± 1.575
0.36	0.25	10.225 ± 0.097	15.383 ± 0.244	10.299 ± 0.101	0.994 ± 0.071	102.248 ± 0.967	153.833 ± 2.443	57.213 ± 3.430	95.659 ± 2.012
0.4	0.25	10.312 ± 0.080	14.708 ± 0.239	10.389 ± 0.085	1.047 ± 0.066	103.118 ± 0.802	147.083 ± 2.392	55.038 ± 2.992	93.591 ± 2.619
0.44	0.25	10.574 ± 0.118	13.675 ± 0.182	10.665 ± 0.124	1.272 ± 0.089	105.736 ± 1.179	136.750 ± 1.815	44.857 ± 3.870	83.054 ± 3.752
0.48	0.25	10.485 ± 0.126	13.717 ± 0.212	10.571 ± 0.132	1.207 ± 0.082	104.850 ± 1.257	137.167 ± 2.125	46.936 ± 3.045	86.776 ± 4.291
0.52	0.25	10.610 ± 0.082	14.233 ± 0.178	10.703 ± 0.087	1.302 ± 0.068	106.099 ± 0.821	142.333 ± 1.775	43.384 ± 3.048	82.209 ± 3.147
0.56	0.25	10.811 ± 0.063	14.042 ± 0.183	10.899 ± 0.065	1.372 ± 0.046	108.108 ± 0.629	140.417 ± 1.832	38.622 ± 2.055	77.277 ± 2.424
0.6	0.25	10.920 ± 0.116	14.192 ± 0.183	11.012 ± 0.121	1.461 ± 0.094	109.198 ± 1.159	141.917 ± 1.832	34.439 ± 2.938	74.134 ± 5.303
0.64	0.25	10.896 ± 0.097	14.267 ± 0.235	10.985 ± 0.102	1.433 ± 0.080	108.964 ± 0.972	142.667 ± 2.348	35.022 ± 3.218	76.090 ± 5.001
0.68	0.25	10.810 ± 0.071	14.317 ± 0.190	10.898 ± 0.076	1.376 ± 0.064	108.102 ± 0.706	143.167 ± 1.899	37.242 ± 2.753	80.812 ± 4.960
0.72	0.25	10.490 ± 0.086	13.933 ± 0.130	10.565 ± 0.090	1.151 ± 0.063	104.902 ± 0.861	139.333 ± 1.303	44.798 ± 3.730	94.032 ± 1.995
0.76	0.25	10.788 ± 0.082	14.208 ± 0.243	10.875 ± 0.086	1.365 ± 0.067	107.881 ± 0.821	142.083 ± 2.429	36.075 ± 2.555	82.830 ± 5.815
0.8	0.25	10.337 ± 0.138	13.692 ± 0.173	10.406 ± 0.140	1.073 ± 0.066	103.367 ± 1.375	136.917 ± 1.730	49.591 ± 5.975	95.725 ± 0.392
0.28	0.3	9.845 ± 0.055	16.117 ± 0.276	9.911 ± 0.058	0.841 ± 0.060	98.453 ± 0.546	161.167 ± 2.758	69.679 ± 5.574	97.705 ± 0.148
0.32	0.3	9.912 ± 0.061	15.342 ± 0.268	9.971 ± 0.063	0.810 ± 0.041	99.123 ± 0.613	153.417 ± 2.678	70.243 ± 3.403	97.661 ± 0.160
0.36	0.3	10.188 ± 0.072	14.900 ± 0.209	10.258 ± 0.075	0.938 ± 0.054	101.882 ± 0.720	149.000 ± 2.089	60.511 ± 3.310	96.750 ± 0.516
0.4	0.3	9.966 ± 0.077	14.767 ± 0.161	10.027 ± 0.080	0.848 ± 0.036	99.662 ± 0.769	147.667 ± 1.614	67.796 ± 3.926	97.436 ± 0.157
0.44	0.3	10.402 ± 0.059	14.067 ± 0.235	10.471 ± 0.061	1.017 ± 0.048	104.024 ± 0.586	140.667 ± 2.348	56.546 ± 2.134	94.656 ± 1.604
0.48	0.3	10.498 ± 0.072	14.308 ± 0.193	10.575 ± 0.073	1.122 ± 0.043	104.984 ± 0.715	143.083 ± 1.929	51.505 ± 2.155	91.844 ± 1.844
0.52	0.3	10.378 ± 0.051	14.042 ± 0.131	10.450 ± 0.054	1.057 ± 0.044	103.778 ± 0.508	140.417 ± 1.311	53.450 ± 2.493	94.677 ± 1.011
0.56	0.3	10.257 ± 0.075	13.408 ± 0.219	10.319 ± 0.078	0.979 ± 0.044	102.567 ± 0.751	134.083 ± 2.193	56.775 ± 2.994	96.625 ± 0.341
0.6	0.3	10.547 ± 0.063	14.258 ± 0.202	10.625 ± 0.067	1.187 ± 0.056	105.473 ± 0.627	142.583 ± 2.021	43.116 ± 2.518	92.138 ± 2.846
0.64	0.3	10.529 ± 0.086	13.658 ± 0.173	10.604 ± 0.089	1.174 ± 0.060	105.289 ± 0.863	136.583 ± 1.730	43.748 ± 3.005	92.446 ± 2.956
0.68	0.3	10.252 ± 0.108	13.342 ± 0.211	10.315 ± 0.113	0.988 ± 0.075	102.520 ± 1.084	133.417 ± 2.109	57.915 ± 6.723	95.088 ± 0.947
0.72	0.3	10.268 ± 0.127	13.192 ± 0.291	10.328 ± 0.132	0.981 ± 0.083	102.675 ± 1.275	131.917 ± 2.906	56.353 ± 7.553	96.249 ± 0.773
0.76	0.3	10.246 ± 0.158	14.375 ± 0.341	10.316 ± 0.165	0.988 ± 0.113	102.455 ± 1.580	143.750 ± 3.415	60.559 ± 8.694	95.229 ± 2.131
0.8	0.3	9.844 ± 0.139	12.308 ± 0.452	9.891 ± 0.152	0.802 ± 0.130	98.440 ± 1.390	123.083 ± 4.522	69.004 ± 8.701	98.653 ± 1.739
0.28	0.35	9.599 ± 0.149	15.058 ± 0.555	9.657 ± 0.156	0.908 ± 0.054	95.995 ± 1.495	150.583 ± 5.551	62.406 ± 3.975	98.027 ± 0.164
0.32	0.35	9.600 ± 0.072	15.408 ± 0.227	9.657 ± 0.073	0.886 ± 0.020	95.998 ± 0.717	154.083 ± 2.275	64.161 ± 1.615	98.327 ± 0.174
0.36	0.35	9.587 ± 0.054	15.608 ± 0.396	9.645 ± 0.056	0.885 ± 0.031	95.866 ± 0.535	156.083 ± 3.965	65.126 ± 1.789	98.341 ± 0.149
0.4	0.35	10.052 ± 0.044	15.308 ± 0.168	10.126 ± 0.047	0.957 ± 0.047	100.525 ± 0.444	153.083 ± 1.676	60.564 ± 3.209	97.502 ± 0.084
0.44	0.35	10.075 ± 0.065	14.392 ± 0.183	10.146 ± 0.067	0.971 ± 0.046	100.750 ± 0.652	143.917 ± 1.832	58.403 ± 3.392	97.231 ± 0.190
0.48	0.35	10.264 ± 0.039	14.492 ± 0.162	10.343 ± 0.041	1.081 ± 0.045	102.641 ± 0.390	144.917 ± 1.621	51.960 ± 2.781	96.164 ± 0.703
0.52	0.35	10.201 ± 0.069	14.767 ± 0.183	10.274 ± 0.074	0.976 ± 0.060	102.009 ± 0.692	147.667 ± 1.826	59.383 ± 4.685	96.611 ± 0.317
0.56	0.35	10.282 ± 0.082	14.092 ± 0.131	10.351 ± 0.084	1.023 ± 0.051	102.821 ± 0.823	140.917 ± 1.311	54.842 ± 4.363	96.452 ± 0.292
0.6	0.35	10.377 ± 0.043	14.367 ± 0.235	10.453 ± 0.046	1.094 ± 0.039	103.765 ± 0.431	143.667 ± 2.348	50.281 ± 2.892	95.386 ± 1.399
0.64	0.35	10.235 ± 0.105	14.217 ± 0.195	10.307 ± 0.108	1.014 ± 0.074	102.355 ± 1.046	142.167 ± 1.946	55.923 ± 5.645	96.350 ± 0.748
0.68	0.35	10.347 ± 0.048	13.958 ± 0.207	10.417 ± 0.050	1.050 ± 0.051	103.469 ± 0.483	139.583 ± 2.065	51.890 ± 4.413	96.132 ± 0.645
0.72	0.35	10.302 ± 0.094	12.983 ± 0.212	10.362 ± 0.096	0.997 ± 0.062	103.016 ± 0.941	129.833 ± 2.125	55.738 ± 5.022	96.157 ± 0.500
0.76	0.35	9.934 ± 0.057	12.458 ± 0.207	9.989 ± 0.062	0.884 ± 0.062	99.339 ± 0.565	124.583 ± 2.065	64.163 ± 4.380	98.230 ± 1.150
0.8	0.35	9.747 ± 0.074	13.325 ± 0.308	9.811 ± 0.080	0.937 ± 0.074	97.473 ± 0.743	133.250 ± 3.079	62.650 ± 6.912	95.628 ± 0.822
0.28	0.4	9.555 ± 0.164	14.592 ± 0.507	9.606 ± 0.171	0.868 ± 0.057	95.549 ± 1.642	145.917 ± 5.071	64.832 ± 4.177	98.099 ± 0.133
0.32	0.4	9.563 ± 0.061	14.858 ± 0.312	9.618 ± 0.063	0.871 ± 0.044	95.633 ± 0.605	148.583 ± 3.118	64.261 ± 2.867	98.242 ± 0.146
0.36	0.4	9.462 ± 0.081	14.100 ± 0.280	9.508 ± 0.086	0.848 ± 0.031	94.623 ± 0.808	141.000 ± 2.796	63.630 ± 1.989	98.314 ± 0.280
0.4	0.4	9.771 ± 0.065	13.833 ± 0.253	9.827 ± 0.069	0.846 ± 0.044	97.709 ± 0.649	138.333 ± 2.535	66.469 ± 3.101	97.490 ± 0.184
0.44	0.4	9.720 ± 0.069	14.683 ± 0.572	9.776 ± 0.073	0.812 ± 0.043	97.196 ± 0.689	146.833 ± 5.718	70.339 ± 2.937	97.719 ± 0.216
0.48	0.4	9.988 ± 0.062	15.050 ± 0.183	10.054 ± 0.066	0.880 ± 0.053	99.881 ± 0.619	150.500 ± 1.834	66.554 ± 4.898	97.390 ± 0.145
0.52	0.4	10.033 ± 0.067	14.242 ± 0.294	10.100 ± 0.071	0.928 ± 0.049	100.335 ± 0.666	142.417 ± 2.937	61.615 ± 4.874	97.226 ± 0.259
0.56	0.4	9.887 ± 0.061	12.717 ± 0.072	9.934 ± 0.063	0.785 ± 0.040	98.869 ± 0.614	127.167 ± 0.718	70.987 ± 3.280	97.739 ± 0.337
0.6	0.4	10.183 ± 0.094	13.250 ± 0.188	10.246 ± 0.098	0.982 ± 0.061	101.830 ± 0.937	132.500 ± 1.883	55.986 ± 5.950	97.021 ± 0.670
0.64	0.4	9.919 ± 0.063	12.717 ± 0.217	9.969 ± 0.065	0.822 ± 0.053	99.187 ± 0.631	127.167 ± 2.167	68.972 ± 3.842	97.848 ± 0.862
0.68	0.4	10.126 ± 0.072	13.258 ± 0.250	10.185 ± 0.079	0.928 ± 0.082	101.256 ± 0.724	132.583 ± 2.503	61.518 ± 6.779	96.483 ± 0.709
0.72	0.4	9.929 ± 0.071	13.117 ± 0.248	9.987 ± 0.073	0.873 ± 0.039	99.289 ± 0.712	131.167 ± 2.480	68.619 ± 3.366	95.527 ± 0.397
0.76	0.4	9.381 ± 0.126	12.975 ± 0.299	9.430 ± 0.127	0.950 ± 0.056	93.810 ± 1.261	129.750 ± 2.989	55.570 ± 5.655	97.493 ± 0.539
0.8	0.4	NA *							
0.28	0.45	9.328 ± 0.123	13.642 ± 0.332	9.367 ± 0.127	0.885 ± 0.073	93.283 ± 1.233	136.417 ± 3.315	60.029 ± 3.854	98.599 ± 0.210
0.32	0.45	9.520 ± 0.091	14.458 ± 0.312	9.571 ± 0.095	0.871 ± 0.024	95.197 ± 0.911	144.583 ± 3.118	63.124 ± 2.242	98.252 ± 0.141
0.36	0.45	9.722 ± 0.063	13.942 ± 0.202	9.777 ± 0.066	0.858 ± 0.034	97.217 ± 0.628	139.417 ± 2.021	66.294 ± 2.458	97.910 ± 0.109
0.4	0.45	9.666 ± 0.113	13.600 ± 0.241	9.714 ± 0.114	0.799 ± 0.026	96.661 ± 1.132	136.000 ± 2.412	69.866 ± 2.368	97.768 ± 0.121
0.44	0.45	9.606 ± 0.058	12.800 ± 0.245	9.652 ± 0.060	0.821 ± 0.041	96.064 ± 0.580	128.000 ± 2.449	66.300 ± 2.981	98.472 ± 0.243
0.48	0.45	9.949 ± 0.076	13.617 ± 0.153	10.003 ± 0.077	0.839 ± 0.062	99.486 ± 0.756	136.167 ± 1.528	68.497 ± 4.477	97.363 ± 0.140
0.52	0.45	9.810 ± 0.061	13.050 ± 0.220	9.862 ± 0.062	0.832 ± 0.034	98.098 ± 0.613	130.500 ± 2.195	68.859 ± 2.395	97.754 ± 0.361
0.56	0.45	9.889 ± 0.048	12.775 ± 0.191	9.939 ± 0.050	0.803 ± 0.041	98.893 ± 0.485	127.750 ± 1.913	70.355 ± 2.681	97.942 ± 0.386
0.6	0.45	9.930 ± 0.081	13.233 ± 0.156	9.985 ± 0.086	0.868 ± 0.071	99.298 ± 0.813	132.333 ± 1.557	65.873 ± 4.825	97.374 ± 0.620
0.64	0.45	9.982 ± 0.082	12.717 ± 0.217	10.030 ± 0.085	0.799 ± 0.064	99.821 ± 0.815	127.167 ± 2.167	70.961 ± 4.393	97.629 ± 0.702
0.68	0.45	9.584 ± 0.058	13.083 ± 0.185	9.638 ± 0.060	0.877 ± 0.071	95.843 ± 0.581	130.833 ± 1.850	63.093 ± 3.903	97.522 ± 0.306
0.72	0.45	9.497 ± 0.136	13.292 ± 0.387	9.551 ± 0.138	0.938 ± 0.053	94.971 ± 1.358	132.917 ± 3.872	60.977 ± 4.588	96.373 ± 0.315
0.76	0.45	NA *							
0.8	0.45	NA *							
0.28	0.5	9.134 ± 0.121	13.858 ± 0.387	9.168 ± 0.123	0.992 ± 0.121	91.340 ± 1.214	138.583 ± 3.872	53.241 ± 9.172	98.665 ± 0.214
0.32	0.5	9.351 ± 0.073	14.075 ± 0.171	9.395 ± 0.075	0.892 ± 0.031	93.511 ± 0.729	140.750 ± 1.712	60.020 ± 2.106	98.431 ± 0.115
0.36	0.5	9.465 ± 0.033	14.017 ± 0.147	9.511 ± 0.034	0.847 ± 0.015	94.647 ± 0.325	140.167 ± 1.467	64.187 ± 1.254	98.332 ± 0.122
0.4	0.5	9.549 ± 0.046	13.742 ± 0.202	9.595 ± 0.048	0.799 ± 0.023	95.490 ± 0.459	137.417 ± 2.021	68.981 ± 1.433	97.969 ± 0.154
0.44	0.5	9.352 ± 0.057	12.658 ± 0.231	9.389 ± 0.059	0.826 ± 0.026	93.523 ± 0.574	126.583 ± 2.314	62.935 ± 1.547	99.007 ± 0.318
0.48	0.5	9.565 ± 0.052	12.850 ± 0.220	9.605 ± 0.054	0.750 ± 0.057	95.650 ± 0.518	128.500 ± 2.195	69.523 ± 3.184	98.468 ± 0.305
0.52	0.5	9.425 ± 0.036	12.350 ± 0.138	9.463 ± 0.037	0.806 ± 0.033	94.250 ± 0.363	123.500 ± 1.382	64.149 ± 2.680	99.128 ± 0.340
0.56	0.5	9.747 ± 0.104	13.050 ± 0.232	9.791 ± 0.108	0.755 ± 0.065	97.471 ± 1.044	130.500 ± 2.316	72.900 ± 3.067	98.170 ± 0.288
0.6	0.5	9.557 ± 0.041	13.733 ± 0.320	9.603 ± 0.043	0.774 ± 0.041	95.572 ± 0.412	137.333 ± 3.200	69.101 ± 2.432	97.537 ± 0.262
0.64	0.5	9.264 ± 0.031	12.442 ± 0.144	9.304 ± 0.032	0.921 ± 0.027	92.643 ± 0.306	124.417 ± 1.443	56.401 ± 2.601	99.419 ± 0.232
0.68	0.5	9.397 ± 0.127	13.625 ± 0.218	9.454 ± 0.130	0.955 ± 0.068	93.974 ± 1.268	136.250 ± 2.179	57.292 ± 5.323	95.886 ± 0.347
0.72	0.5	NA *							
0.76	0.5	NA *							
0.8	0.5	NA *							

NA *—not applicable.

**Table A2:** Summary of the results of the experiment carried out according to the second post-measurement scenario (set values: Force in *X*-axis in the range from 1 N to 10 N; Speed Limits = 0.001 m/s; Distances: 0.55 m, 0.61 m, 0.65 m, 0.70 m, 0.75 m, 0.80 m, 0.85 m, 0.90 m, and 0.95 m).

Force [N]	Distance [m]	Mean Force ± Std [N]	Mean Max ± Std [N]	Mean RMSE ± Std [N]	Mean MAE ± Std [N]	Mean Rate ± Std [%]	Mean Overshoot ± Std [%]	Mean P10 ± Std [%]	Mean P20 ± Std [%]
1	0.55	1.045 ± 0.159	1.051 ± 0.168	1.051 ± 0.168	0.120 ± 0.128	104.517 ± 15.889	118.516 ± 31.038	81.310 ± 27.170	89.982 ± 20.039
2	0.55	1.912 ± 0.223	1.917 ± 0.231	1.917 ± 0.231	0.225 ± 0.126	95.587 ± 11.141	105.605 ± 21.114	60.732 ± 34.231	94.856 ± 15.333
3	0.55	2.988 ± 0.459	3.001 ± 0.477	3.001 ± 0.477	0.439 ± 0.242	99.586 ± 15.286	112.606 ± 25.905	42.868 ± 38.399	84.891 ± 27.010
4	0.55	3.453 ± 0.175	3.455 ± 0.179	3.455 ± 0.179	0.567 ± 0.108	86.333 ± 4.377	89.749 ± 7.926	18.053 ± 26.188	99.768 ± 2.293
5	0.55	4.339 ± 0.221	4.342 ± 0.226	4.342 ± 0.226	0.689 ± 0.143	86.778 ± 4.426	91.281 ± 7.927	20.012 ± 25.304	100.000 ± 0.000
6	0.55	5.511 ± 0.516	5.522 ± 0.529	5.522 ± 0.529	0.711 ± 0.295	91.854 ± 8.607	99.712 ± 15.088	40.665 ± 39.144	99.113 ± 3.197
7	0.55	6.479 ± 0.573	6.491 ± 0.585	6.491 ± 0.585	0.770 ± 0.350	92.559 ± 8.184	100.102 ± 12.980	51.843 ± 38.305	100.000 ± 0.000
8	0.55	7.005 ± 0.441	7.011 ± 0.449	7.011 ± 0.449	1.051 ± 0.339	87.565 ± 5.515	92.740 ± 9.389	30.681 ± 31.701	100.000 ± 0.000
9	0.55	7.881 ± 0.486	7.888 ± 0.494	7.888 ± 0.494	1.159 ± 0.420	87.565 ± 5.401	92.769 ± 8.854	31.210 ± 31.190	100.000 ± 0.000
10	0.55	8.656 ± 0.509	8.662 ± 0.517	8.662 ± 0.517	1.372 ± 0.457	86.555 ± 5.089	90.897 ± 8.102	22.542 ± 30.528	100.000 ± 0.000
1	0.61	0.987 ± 0.122	0.990 ± 0.127	0.990 ± 0.127	0.094 ± 0.091	98.700 ± 12.190	106.048 ± 21.602	91.068 ± 19.379	95.770 ± 13.654
2	0.61	1.827 ± 0.167	1.829 ± 0.172	1.829 ± 0.172	0.228 ± 0.103	91.336 ± 8.370	96.778 ± 14.967	52.886 ± 39.369	98.232 ± 10.228
3	0.61	2.676 ± 0.180	2.679 ± 0.185	2.679 ± 0.185	0.363 ± 0.110	89.212 ± 5.989	94.586 ± 11.210	39.201 ± 35.446	98.544 ± 8.911
4	0.61	3.538 ± 0.262	3.543 ± 0.270	3.543 ± 0.270	0.526 ± 0.145	88.462 ± 6.559	93.756 ± 11.753	27.477 ± 32.222	98.887 ± 5.356
5	0.61	4.445 ± 0.359	4.452 ± 0.369	4.452 ± 0.369	0.660 ± 0.191	88.903 ± 7.186	95.104 ± 12.942	28.812 ± 31.516	98.892 ± 5.298
6	0.61	5.066 ± 0.221	5.068 ± 0.225	5.068 ± 0.225	0.953 ± 0.139	84.441 ± 3.682	86.862 ± 6.119	6.121 ± 16.102	99.914 ± 1.387
7	0.61	6.179 ± 0.501	6.187 ± 0.514	6.187 ± 0.514	0.957 ± 0.282	88.269 ± 7.163	93.481 ± 12.075	27.228 ± 32.176	100.000 ± 0.000
8	0.61	7.272 ± 0.679	7.286 ± 0.695	7.286 ± 0.695	0.987 ± 0.407	90.905 ± 8.490	98.000 ± 14.352	42.629 ± 38.353	99.996 ± 0.026
9	0.61	8.259 ± 0.641	8.275 ± 0.657	8.275 ± 0.657	0.957 ± 0.488	91.765 ± 7.127	100.920 ± 13.396	55.717 ± 41.394	100.000 ± 0.000
10	0.61	9.207 ± 0.585	9.221 ± 0.597	9.221 ± 0.597	0.946 ± 0.489	92.071 ± 5.849	103.400 ± 12.011	66.613 ± 36.623	100.000 ± 0.000
1	0.65	0.987 ± 0.110	0.989 ± 0.115	0.989 ± 0.115	0.090 ± 0.080	98.686 ± 11.027	105.465 ± 19.548	91.895 ± 18.859	97.071 ± 11.215
2	0.65	1.907 ± 0.257	1.913 ± 0.269	1.913 ± 0.269	0.241 ± 0.163	95.371 ± 12.873	104.667 ± 23.428	53.139 ± 38.893	95.274 ± 16.091
3	0.65	2.864 ± 0.321	2.872 ± 0.333	2.872 ± 0.333	0.346 ± 0.173	95.455 ± 10.705	105.934 ± 18.571	54.150 ± 36.584	92.181 ± 20.051
4	0.65	3.635 ± 0.330	3.641 ± 0.341	3.641 ± 0.341	0.492 ± 0.164	90.875 ± 8.249	98.457 ± 14.233	38.648 ± 34.651	96.267 ± 11.723
5	0.65	4.521 ± 0.400	4.529 ± 0.411	4.529 ± 0.411	0.626 ± 0.204	90.419 ± 7.994	97.802 ± 13.837	34.503 ± 33.165	97.960 ± 8.511
6	0.65	5.442 ± 0.531	5.453 ± 0.547	5.453 ± 0.547	0.789 ± 0.254	90.703 ± 8.852	97.692 ± 14.926	31.993 ± 32.840	98.806 ± 4.061
7	0.65	6.194 ± 0.453	6.201 ± 0.463	6.201 ± 0.463	0.921 ± 0.243	88.484 ± 6.465	94.154 ± 10.086	28.167 ± 28.901	100.000 ± 0.000
8	0.65	7.230 ± 0.566	7.241 ± 0.579	7.241 ± 0.579	0.965 ± 0.302	90.371 ± 7.076	97.772 ± 11.374	37.465 ± 30.555	100.000 ± 0.000
9	0.65	8.133 ± 0.665	8.143 ± 0.677	8.143 ± 0.677	1.072 ± 0.389	90.363 ± 7.389	96.593 ± 10.862	40.397 ± 34.365	100.000 ± 0.000
10	0.65	9.547 ± 0.451	9.558 ± 0.456	9.558 ± 0.456	0.594 ± 0.421	95.473 ± 4.506	110.400 ± 9.869	85.672 ± 26.108	100.000 ± 0.000
1	0.70	1.210 ± 0.308	1.225 ± 0.329	1.225 ± 0.329	0.257 ± 0.279	121.049 ± 30.785	148.727 ± 56.327	58.081 ± 38.539	70.631 ± 35.369
2	0.70	1.953 ± 0.343	1.963 ± 0.364	1.963 ± 0.364	0.291 ± 0.214	97.650 ± 17.152	108.689 ± 31.706	51.042 ± 35.474	90.406 ± 21.426
3	0.70	3.007 ± 0.494	3.024 ± 0.518	3.024 ± 0.518	0.481 ± 0.257	100.232 ± 16.470	113.617 ± 30.047	36.343 ± 37.290	83.610 ± 27.096
4	0.70	3.912 ± 0.502	3.927 ± 0.520	3.927 ± 0.520	0.522 ± 0.251	97.795 ± 12.543	110.111 ± 22.002	39.210 ± 38.775	88.686 ± 20.683
5	0.70	4.710 ± 0.506	4.719 ± 0.517	4.719 ± 0.517	0.562 ± 0.281	94.197 ± 10.112	106.118 ± 19.688	45.426 ± 39.844	96.069 ± 11.706
6	0.70	5.898 ± 0.425	5.914 ± 0.439	5.914 ± 0.439	0.480 ± 0.339	98.296 ± 7.090	111.825 ± 13.570	65.086 ± 38.668	96.555 ± 7.807
7	0.70	6.625 ± 0.494	6.642 ± 0.507	6.642 ± 0.507	0.650 ± 0.364	94.646 ± 7.054	105.385 ± 13.219	60.397 ± 37.693	99.880 ± 0.613
8	0.70	7.992 ± 0.308	8.016 ± 0.326	8.016 ± 0.326	0.499 ± 0.391	99.903 ± 3.849	116.103 ± 7.314	73.895 ± 32.667	98.857 ± 3.757
9	0.70	8.590 ± 0.504	8.608 ± 0.517	8.608 ± 0.517	0.662 ± 0.453	95.448 ± 5.602	107.836 ± 11.609	71.894 ± 34.628	99.986 ± 0.044
10	0.70	9.485 ± 0.190	9.492 ± 0.190	9.492 ± 0.190	0.564 ± 0.191	94.846 ± 1.905	111.154 ± 5.194	89.948 ± 16.587	100.000 ± 0.000
1	0.75	1.170 ± 0.293	1.182 ± 0.311	1.182 ± 0.311	0.235 ± 0.252	116.987 ± 29.336	140.311 ± 53.918	63.404 ± 38.835	73.748 ± 33.384
2	0.75	1.853 ± 0.193	1.856 ± 0.199	1.856 ± 0.199	0.231 ± 0.109	92.641 ± 9.634	99.276 ± 17.677	53.576 ± 39.348	97.008 ± 12.387
3	0.75	2.852 ± 0.423	2.863 ± 0.443	2.863 ± 0.443	0.430 ± 0.212	95.082 ± 14.095	104.587 ± 25.368	35.191 ± 33.897	90.543 ± 20.672
4	0.75	3.855 ± 0.499	3.870 ± 0.518	3.870 ± 0.518	0.533 ± 0.242	96.381 ± 12.463	108.259 ± 22.472	37.847 ± 36.910	90.325 ± 20.245
5	0.75	4.626 ± 0.536	4.639 ± 0.557	4.639 ± 0.557	0.676 ± 0.255	92.512 ± 10.725	101.206 ± 18.846	31.198 ± 35.557	92.543 ± 17.245
6	0.75	5.692 ± 0.655	5.705 ± 0.671	5.705 ± 0.671	0.718 ± 0.335	94.859 ± 10.915	104.667 ± 18.309	40.727 ± 38.892	91.977 ± 16.676
7	0.75	7.108 ± 0.855	7.134 ± 0.894	7.134 ± 0.894	0.710 ± 0.656	101.542 ± 12.218	119.286 ± 21.293	64.934 ± 39.749	92.339 ± 20.942
8	0.75	7.919 ± 0.565	7.932 ± 0.572	7.932 ± 0.572	0.557 ± 0.375	98.984 ± 7.062	113.897 ± 12.899	73.508 ± 35.389	99.728 ± 0.479
9	0.75	8.268 ± 0.709	8.279 ± 0.720	8.279 ± 0.720	0.915 ± 0.570	91.870 ± 7.873	101.806 ± 15.409	56.481 ± 43.751	99.994 ± 0.031
10	0.75	9.121 ± 0.698	9.130 ± 0.705	9.130 ± 0.705	0.985 ± 0.613	91.205 ± 6.975	102.292 ± 14.956	53.895 ± 43.590	99.975 ± 0.125
1	0.80	0.972 ± 0.096	0.974 ± 0.099	0.974 ± 0.099	0.083 ± 0.068	97.208 ± 9.556	102.117 ± 16.259	94.141 ± 17.615	97.888 ± 11.003
2	0.80	1.791 ± 0.110	1.792 ± 0.113	1.792 ± 0.113	0.228 ± 0.075	89.526 ± 5.480	93.267 ± 10.081	50.044 ± 39.237	99.205 ± 6.839
3	0.80	2.730 ± 0.242	2.735 ± 0.250	2.735 ± 0.250	0.360 ± 0.126	91.003 ± 8.064	98.038 ± 14.792	44.461 ± 36.589	96.881 ± 12.518
4	0.80	3.762 ± 0.431	3.774 ± 0.446	3.774 ± 0.446	0.506 ± 0.215	94.058 ± 10.775	103.589 ± 18.750	38.957 ± 38.867	92.683 ± 15.564
5	0.80	4.408 ± 0.349	4.412 ± 0.357	4.412 ± 0.357	0.685 ± 0.163	88.150 ± 6.977	93.239 ± 11.419	24.147 ± 28.339	99.094 ± 4.464
6	0.80	5.063 ± 0.205	5.065 ± 0.209	5.065 ± 0.209	0.953 ± 0.134	84.389 ± 3.425	86.789 ± 5.929	6.004 ± 15.672	100.000 ± 0.000
7	0.80	6.385 ± 0.522	6.397 ± 0.536	6.397 ± 0.536	0.818 ± 0.307	91.213 ± 7.463	98.893 ± 12.635	45.739 ± 32.023	100.000 ± 0.000
8	0.80	7.383 ± 0.590	7.399 ± 0.605	7.399 ± 0.605	0.880 ± 0.365	92.293 ± 7.373	99.545 ± 12.298	52.170 ± 36.827	100.000 ± 0.000
9	0.80	8.142 ± 0.595	8.154 ± 0.608	8.154 ± 0.608	1.014 ± 0.443	90.463 ± 6.606	97.527 ± 12.194	52.922 ± 39.920	100.000 ± 0.000
10	0.80	8.749 ± 0.566	8.758 ± 0.577	8.758 ± 0.577	1.302 ± 0.494	87.494 ± 5.656	92.634 ± 9.851	32.692 ± 35.418	100.000 ± 0.000
1	0.85	0.983 ± 0.122	0.986 ± 0.127	0.986 ± 0.127	0.104 ± 0.075	98.307 ± 12.159	104.444 ± 22.774	90.310 ± 21.757	92.931 ± 18.372
2	0.85	1.925 ± 0.233	1.931 ± 0.245	1.931 ± 0.245	0.215 ± 0.155	96.268 ± 11.674	106.649 ± 21.432	68.947 ± 32.955	96.235 ± 14.307
3	0.85	2.688 ± 0.259	2.692 ± 0.267	2.692 ± 0.267	0.384 ± 0.155	89.614 ± 8.625	95.066 ± 14.486	37.187 ± 37.195	97.544 ± 12.995
4	0.85	3.555 ± 0.322	3.560 ± 0.331	3.560 ± 0.331	0.539 ± 0.173	88.878 ± 8.044	94.403 ± 14.206	26.180 ± 34.009	97.434 ± 11.479
5	0.85	4.235 ± 0.172	4.236 ± 0.175	4.236 ± 0.175	0.778 ± 0.119	84.699 ± 3.442	87.037 ± 6.091	8.151 ± 17.679	100.000 ± 0.000
6	0.85	5.436 ± 0.462	5.445 ± 0.475	5.445 ± 0.475	0.742 ± 0.216	90.593 ± 7.694	97.608 ± 12.416	36.058 ± 29.116	99.132 ± 3.647
7	0.85	6.406 ± 0.543	6.419 ± 0.558	6.419 ± 0.558	0.823 ± 0.291	91.520 ± 7.763	99.135 ± 12.994	44.005 ± 35.295	99.834 ± 1.025
8	0.85	7.049 ± 0.509	7.057 ± 0.520	7.057 ± 0.520	1.053 ± 0.340	88.115 ± 6.367	93.148 ± 9.919	28.079 ± 31.666	100.000 ± 0.000
9	0.85	7.995 ± 0.613	8.006 ± 0.627	8.006 ± 0.627	1.158 ± 0.402	88.837 ± 6.808	94.123 ± 11.011	34.907 ± 36.284	100.000 ± 0.000
10	0.85	8.736 ± 0.565	8.745 ± 0.577	8.745 ± 0.577	1.334 ± 0.458	87.356 ± 5.646	92.057 ± 9.590	29.291 ± 34.168	100.000 ± 0.000
1	0.90	1.020 ± 0.138	1.023 ± 0.144	1.023 ± 0.144	0.101 ± 0.109	101.959 ± 13.824	111.664 ± 25.766	87.060 ± 23.577	93.871 ± 16.004
2	0.90	1.881 ± 0.217	1.886 ± 0.226	1.886 ± 0.226	0.220 ± 0.139	94.063 ± 10.843	101.841 ± 19.686	62.196 ± 37.026	96.463 ± 14.469
3	0.90	2.735 ± 0.323	2.741 ± 0.335	2.741 0.335	0.410 ± 0.154	91.160 ± 10.752	97.753 ± 19.272	32.935 ± 36.907	94.784 ± 16.639
4	0.90	3.580 ± 0.358	3.585 ± 0.367	3.585 ± 0.367	0.549 ± 0.157	89.497 ± 8.949	94.955 ± 15.385	25.022 ± 32.828	96.637 ± 10.968
5	0.90	4.376 ± 0.348	4.380 ± 0.355	4.380 ± 0.355	0.700 ± 0.216	87.512 ± 6.952	92.327 ± 12.418	21.339 ± 33.661	99.909 ± 0.602
6	0.90	5.359 ± 0.468	5.366 ± 0.478	5.366 ± 0.478	0.769 ± 0.302	89.322 ± 7.803	95.000 ± 13.472	34.305 ± 38.735	99.905 ± 0.702
7	0.90	6.097 ± 0.411	6.102 ± 0.419	6.102 ± 0.419	0.959 ± 0.320	87.102 ± 5.874	91.797 ± 11.075	25.321 ± 38.629	100.000 ± 0.000
8	0.90	7.704 ± 0.376	7.711 ± 0.377	7.711 ± 0.377	0.413 ± 0.325	96.303 ± 4.702	110.982 ± 9.369	86.285 ± 27.318	100.000 ± 0.000
9	0.90	8.227 ± 0.478	8.234 ± 0.482	8.234 ± 0.482	0.810 ± 0.457	91.406 ± 5.317	101.389 ± 10.638	66.302 ± 38.171	100.000 ± 0.000
10	0.90	8.599 ± 0.471	8.602 ± 0.475	8.602 ± 0.475	1.407 ± 0.461	85.994 ± 4.714	90.732 ± 9.389	27.145 ± 39.152	100.000 ± 0.000
1	0.95	1.202 ± 0.502	1.206 ± 0.505	1.206 ± 0.505	0.262 ± 0.475	120.169 ± 50.156	140.989 ± 83.281	75.659 ± 34.849	84.434 ± 32.901
2	0.95	1.987 ± 0.551	1.989 ± 0.554	1.989 ± 0.554	0.360 ± 0.417	99.348 ± 27.559	107.500 ± 42.525	46.000 ± 41.820	88.473 ± 31.638
3	0.95	3.156 ± 0.694	3.161 ± 0.697	3.161 ± 0.697	0.569 ± 0.428	105.197 ± 23.126	121.282 ± 38.325	38.658 ± 39.397	71.044 ± 44.373
4	0.95	3.541 ± 0.455	3.543 ± 0.457	3.543 ± 0.457	0.638 ± 0.100	88.527 ± 11.377	93.131 ± 19.592	6.253 ± 13.658	95.442 ± 14.412
5	0.95	4.338 ± 0.433	4.339 ± 0.434	4.339 ± 0.434	0.783 ± 0.114	86.762 ± 8.654	90.382 ± 14.837	7.262 ± 17.753	99.527 ± 1.938
6	0.95	6.544 ± 0.719	6.550 ± 0.718	6.550 ± 0.718	0.867 ± 0.210	109.066 ± 11.975	123.778 ± 16.732	20.822 ± 25.816	84.797 ± 13.562
7	0.95	6.041 ± 0.537	6.042 ± 0.539	6.042 ± 0.539	1.048 ± 0.333	86.298 ± 7.678	89.865 ± 12.568	15.799 ± 34.146	99.984 ± 0.124
8	0.95	7.381 ± 0.844	7.384 ± 0.845	7.384 ± 0.845	0.954 ± 0.417	92.261 ± 10.547	98.500 ± 15.191	39.304 ± 44.202	99.995 ± 0.028
9	0.95	8.700 ± 0.695	8.703 ± 0.696	8.703 ± 0.696	0.563 ± 0.514	96.664 ± 7.722	105.139 ± 10.664	72.265 ± 42.705	100.000 ± 0.000
10	0.95	9.483 ± 0.667	9.487 ± 0.668	9.487 ± 0.668	0.605 ± 0.600	94.833 ± 6.671	101.313 ± 9.236	76.005 ± 38.265	100.000 ± 0.000
